# VarSCAT: A computational tool for sequence context annotations of genomic variants

**DOI:** 10.1371/journal.pcbi.1010727

**Published:** 2023-08-11

**Authors:** Ning Wang, Sofia Khan, Laura L. Elo

**Affiliations:** 1 Turku Bioscience Centre, University of Turku and Åbo Akademi University, Turku, Finland; 2 InFLAMES Research Flagship Center, University of Turku, Turku, Finland; 3 Institute of Biomedicine, University of Turku, Turku, Finland; Thomas Jefferson University, UNITED STATES

## Abstract

The sequence contexts of genomic variants play important roles in understanding biological significances of variants and potential sequencing related variant calling issues. However, methods for assessing the diverse sequence contexts of genomic variants such as tandem repeats and unambiguous annotations have been limited. Herein, we describe the Variant Sequence Context Annotation Tool (VarSCAT) for annotating the sequence contexts of genomic variants, including breakpoint ambiguities, flanking bases of variants, wildtype/mutated DNA sequences, variant nomenclatures, distances between adjacent variants, tandem repeat regions, and custom annotation with user customizable options. Our analyses demonstrate that VarSCAT is more versatile and customizable than the currently available methods or strategies for annotating variants in short tandem repeat (STR) regions or insertions and deletions (indels) with breakpoint ambiguity. Variant sequence context annotations of high-confidence human variant sets with VarSCAT revealed that more than 75% of all human individual germline and clinically relevant indels have breakpoint ambiguities. Moreover, we illustrate that more than 80% of human individual germline small variants in STR regions are indels and that the sizes of these indels correlated with STR motif sizes. VarSCAT is available from https://github.com/elolab/VarSCAT.

## Author summary

The sequence contexts of genomic variants have significant impacts on the biological and technical aspects. These sequence contexts, such as tandem repeats or nearby insertions and deletions (indels), may increase the mutation rate of a region compared to other genome regions. Besides, variants in specific sequence contexts like short tandem repeats (STRs) may also have distinguished biological consequences, which can lead to certain human diseases and thus they may be used as biomarkers for disease diagnosis and treatments. Moreover, potential ambiguous variant representations such as equivalent or redundant indels are also related to their sequence contexts, which may complicate variant harmonization from different sources. Our previous study demonstrated that more than half of false positive indel calls detected through next generation sequencing data were related to simple repeats. Thus, the sequence contexts of genomic variants are important and cannot be ignored. However, the current methods or strategies for assessing the sequence contexts of genomic variants are limited and not feasible to use. Here, we developed a computational tool VarSCAT for sequence context annotation of genomic variants. Although the current version of VarSCAT cannot distinguish whether the genomic variants called by variant calling tools are true or false, our tool provides diverse sequence context annotations, allowing users to further explore their variants of interest. By applying VarSCAT to high confidence human variant sets, we demonstrate the influence of sequence context of genomic variants and emphasize the importance of sequence context assessment.

## Introduction

**Fig 1:**
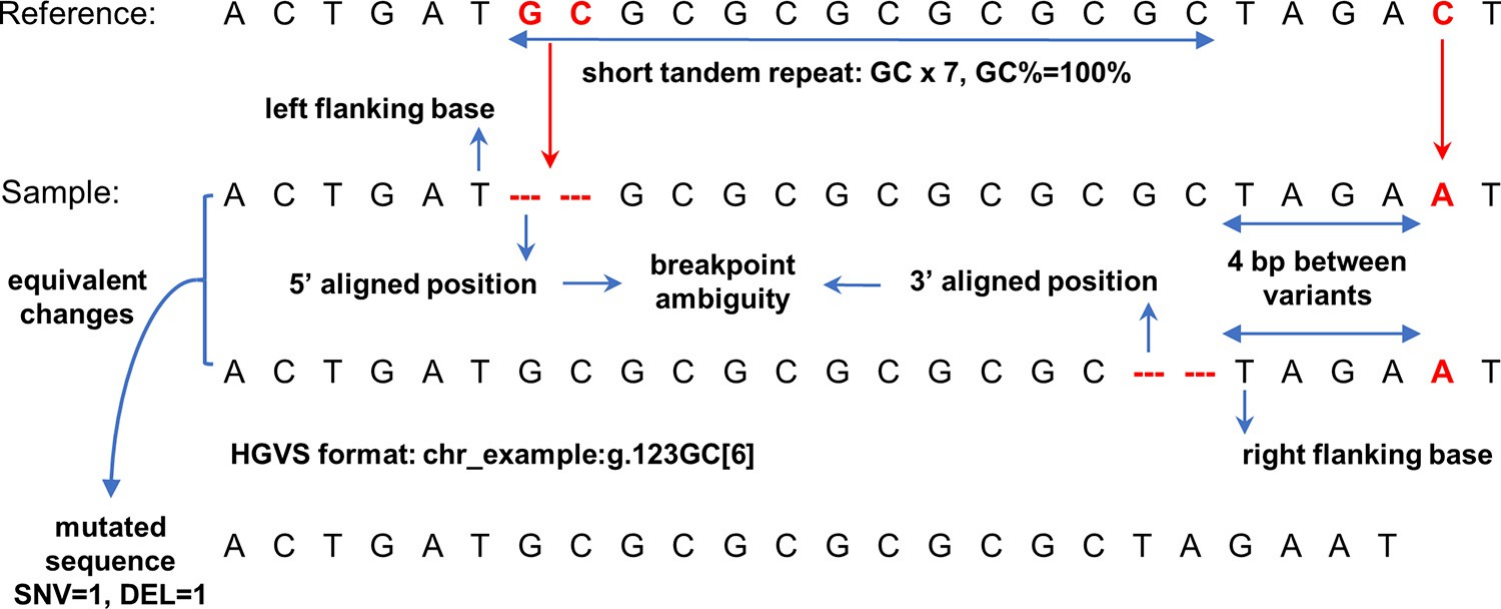
The illustration of sequence contexts of a genomic variant. The example shows the reference and sample sequences where a dinucleotide GC is deleted at “chr_example”, position 123. The deletion is located in a short tandem repeat region, which is a dinucleotide repeat motif GC with a copy number of seven. The GC content (GC%) of the short tandem repeat region is 100%. The short tandem repeat can result in the deletion having multiple possible representations but all lead to an equivalent change. This issue is also known as the breakpoint ambiguity, which indicates the exact breakpoint of a variant is impossible to be confidently identified. The Human Genome Variation Society (HGVS) recommends describing different types of variants with specific roles and formats. The left and right flanking bases of the deletion are marked based on these equivalent deletions on the sequence. There is also a single nucleotide substitution located in the 3’ direction of this deletion. The distance between two variants is 4 bp, which is also determined from on these equivalent deletions. The mutated sequence can be determined by considering all the variants within the region, which in the above example are one single nucleotide variant (SNV) and one deletion (DEL). The variant annotations related to the sequence contexts are shown in bold text.

Genomic variants can influence the fundamental biological processes. Germline variants, which occur in germ cells and can be transmitted to subsequent generations, are the major source of heritable genetic variation. Somatic variants, which occur in any cells except germ cells, can only be transmitted to their daughter cells [1]. These genomic variants may result in gain or loss of functions of their encoded proteins and cause diseases, such as cancers, or show associations with certain phenotypes through gene regulation networks [2,3]. The sequence contexts of genomic variants can have biological and technical influences on the properties of variants (Fig 1). Several studies have shown that the mutation rate of variants is affected by nearby nucleotide patterns and genomic features such as GC contents and CpG islands [4–6]. Another study illustrated that the single nucleotide mutation rate increased when nearby insertions and deletions (indels) were present [7]. Short tandem repeats (STRs), which are mainly caused by the DNA strand slippage and that compose approximately 3% of the human genome, are known as important sequence context features of genomic variants [8,9]. In humans, STR regions have relatively high mutation rates compared with single nucleotides, making them among the fastest-evolving DNA sequences [10–12]. The evolutionary mutational pattern of STRs usually increases or decreases by one repeat motif at a time, but the pattern can also be complicated and heterogeneous [13]. Variants in STR regions may also play important roles in the molecular and cellular functions associated with human health and diseases. For examples, the expansion of a CAG trinucleotide repeat in the *HTT* gene can causes Huntington’s disease, and an increased copy number of a CGG trinucleotide repeat in the *FMR1* gene can causes fragile X syndrome [14–16]. Microsatellite instability is found in tumor tissues of many cancer types, containing STR mutations caused by impaired DNA repair system. Microsatellite instability can be used as biomarkers for cancer diagnosis and treatment [17–20]. *In vitro* research has demonstrated that the sequence context of frame-shift indels in STR regions may promote their tolerance via bypass of transcriptional or translational errors [21].

The sequence contexts of genomic variants can also cause difficulties in next generation sequencing (NGS) data analysis, such as breakpoint ambiguity of indel calling [22,23]. Breakpoint ambiguity is caused by microhomological subsequences surrounding an indel site, which create an equivalent region for the indel, making it impossible to identify the exact breakpoint position of the indel [22,23]. Breakpoint ambiguities may cause problems for downstream annotations. For example, the Human Genome Variation Society (HGVS) recommends a 3’-aligned position for variant nomenclature with respect to the transcript sequence orientation [24]. The indels in the equivalent region may lead to redundant indels in databases [25,26]. Krawitz et al. 2010 illustrated that indel breakpoint ambiguities can affect the sensitivity of indel calling and suggested the unambiguous annotation of an indel, which should have a single coordinate and an equivalent indel region, depending on its sequence context [22]. Shrestha et al. 2018 showed that 40% of deletions ≥ 32 bp in the human genome cannot be identified with unique positions by alignments of 100 bp sequencing reads [23]. In our previous study, we found that more than half of the false positive indels detected by a variety of variant calling methods using NGS data were located in the simple repeats [27]. These observations highlight that the indel breakpoint ambiguity caused by similar local sequence contexts cannot be ignored. The low complexity and highly similar sequence contexts of STRs may also cause technical problems in NGS. The relatively short reads of the Illumina sequencing platform [28] cannot fully resolve long STR regions because the repeat regions may be longer than the length of the reads [29]. Although the single molecule real-time sequencing platforms, such as those from Pacific Biosystems [30] and Oxford Nanopore Technologies [31], have longer read lengths, which can successfully span large regions of STRs, additional costs and requirement of fresh samples may limit their further application [29].

A variety of methods and resources can be utilized to assess the sequence contexts of variants in the human genome (Table 1). The sequence contexts can be assessed by analyzing the reference sequence or calling variants in specific sequence contexts such as STR regions. For example, Benson et al. 1999 developed the Tandem Repeats Finder (TRF) programme by applying a probabilistic model to locate STRs with DNA sequences in FASTA format [32]. Similarly, RepeatMasker is a program that screens DNA sequences in FASTA format for repetitive and low complexity DNA sequences [33]. The ‘Simple Repeats’ and the ‘RepeatMasker’ tracks of the current version of the University of California Santa Cruz’s (UCSC’s) Genome Browser were created using TRF and RepeatMasker, respectively [34]. Krait is a computational tool for detecting different types of STRs with user-defined parameters from a DNA sequence in FASTA format [35]. Tools including TRF, RepeatMakser, or Krait can be used to generate STR annotations of the reference sequence. These STR annotations can be then used for annoating genomic variants with other annoation tools such as ANNOVAR [36]. The ‘TandemRepeat’ function of the Genome Annotation Toolkit (GATK) variant annotation module can annotate variants located in perfect STRs from a input Variant Call Format (VCF) file [37]. HipSTR [38], STRetch [39], and GangSTR [40] are computational STR calling tools that take either short-read sequencing FASTQ files or read alignment files as the inputs to analyze tandem repeats in pre-selected STR regions. These pre-selected STR regions may require prior knowledge from users to know which STR regions should be selected and additional steps are needed for producing them in specific formats required by the STR calling tools. Besides STR sequence context, other types of sequence contexts may also be assessed by other tools. UPS-indel is a web tool with an additional command-line interface that uses a universal positioning system to mark potential breakpoint ambiguities and determine the unique coordinates of indels from VCF input files [26]. SeqTailor is a web server for extracting wildtype/mutated DNA or protein sequences with reference and alternative alleles directly from VCF input files, which is useful for retrieving information about the sequence contexts of variant sites [41]. The Variant Tools software has several functions for variant analysis, one of which can output flanking bases of reference and alternative alleles from VCF or custom-format input files [42]. VariantValidator is a tool for validation, mapping and formatting of sequence variant descriptions. One of its functions is automatic conversion of variants in VCF format into the HGVS format and vice-versa [43].

**Table 1:** Examples of tools which have functions for annotating the sequence context of genomic variants, including breakpoint ambiguity, flanking bases of genomic variants, wildtype/mutated sequences, HGVS nomenclature, nearby variants and short tandem repeats (STR). Note that primary functions of the listed tools may not be specifically designed for annotating sequence context of genomic variants. Many listed tools also have diverse functions on other aspects.

Tools	Breakpoint ambiguity	Variant flanking bases	Wildtype/mutated DNA sequences	HGVS	Nearby variant	STR	Additional information
VarSCAT	**X**	**X**	**X**	DNA level only	**X**	**X**	Variant sequence annotation tool
Tandem Repeat Finder [32]						**X**	Annotate STRs in FASTA
RepeatMasker [33]						**X**	Annotate STRs in FASTA
Krait [35]						**X**	Annotate STRs in FASTA
HipSTR [38]						**X**	STR calling tool for NGS
STRetch [39]						**X**	STR expansion calling tool for NGS
GangSTR [40]						**X**	STR calling tool for NGS
GATK TandemRepeat [37]						**X**	Annotate variants as STRs in VCF
UPS-indel [26]	**X**						Annotate breakpoint ambiguity in VCF
SeqTailor [41]			**X**				Extract wildtype/mutated sequences with VCF and FASTA
Variant tools [42]		**X**	Wildtype only				A tool for manipulation, annotation, and analysis of genomic variants
VariantValidtor [43]				**X**			A tool for validation, mapping and formatting of sequence variants

However, currently, no tool can comprehensively annotate the sequence contexts of variants directly from VCF files in a high-throughput manner. For example, the existing STR annotation methods, using STR locations in BED format to annotate variants in a VCF file, may require several tools or steps; thus, a computational pipeline may be needed to annotate variants with pre-selected STR locations. Also, due to the choice of parameters, these methods may only focus on certain types of STRs, limiting the further analysis, and some tools, such as SeqTailor and Variant Tools, have functions that visualize sequence contexts around variant sites but cannot give information about local sequence complexity. Herein, we present VarSCAT, which is a variant sequence context annotation tool with various functions for studying the sequence contexts around variants and annotating variants with breakpoint ambiguities, flanking bases of variants, wildtype/mutated DNA sequences, HGVS nomenclature, distances between adjacent variants, tandem repeat regions, and custom annotations.

## Results

### An overview of VarSCAT

**Fig 2:**
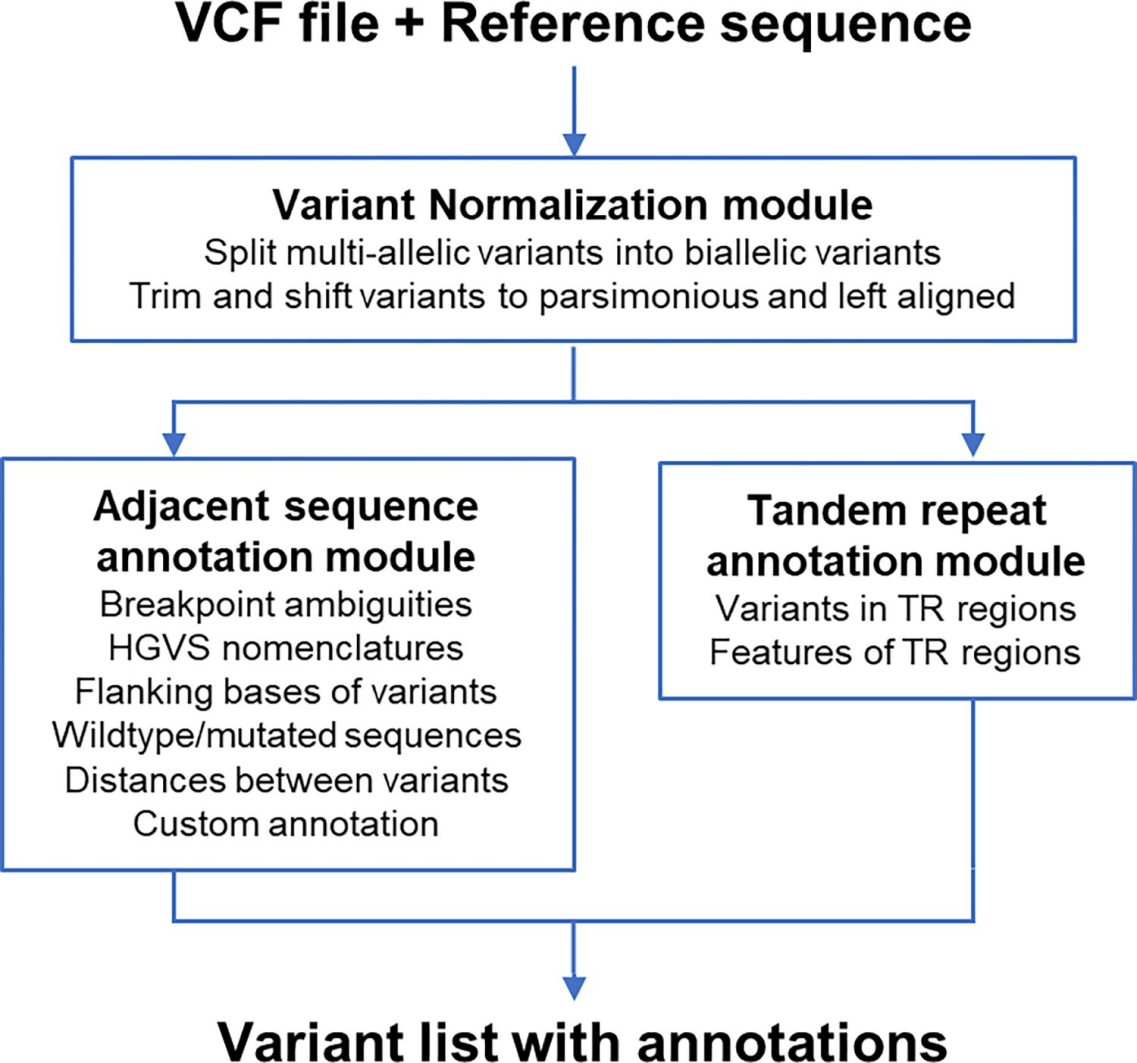
VarSCAT workflow. The input VCF file is first passed to the variant normalization module of VarSCAT, from which the essential information, including positions, reference/alternative alleles, identifiers, and genotypes of variants are extracted. This module can split any potential multiallelic variants into biallelic variants and then normalizes all input variants as parsimonious and left aligned. The output of the variant normalization module is passed to an adjacent sequence annotation module and a tandem repeat (TR) annotation module. The adjacent sequence annotation module can be used to annotate the breakpoint ambiguities, flanking bases of variants, wildtype/mutated DNA sequences, HGVS nomenclature, distances between adjacent variants, and custom annotations. The tandem repeat annotation module can annotate tandem repeat regions of input variants.

We developed a command-line-based computational tool VarSCAT for annotating the sequence contexts of genomic variants. VarSCAT takes a VCF file and a reference sequence as inputs to provide information about the sequence contexts of variants with single-line commands (Fig 2). The variant normalization module is a pre-processing module which first splits any potential multiallelic variants into biallelic variants and then normalizes all input variants as parsimonious and left aligned (details in “Methods and Materials”). The adjacent sequence annotation module is able to output breakpoint ambiguity and the coordinates of affected regions of variants, the HGVS nomenclature of variants based on the DNA level, distances between adjacent variants, and the flanking bases of both the reference and alternative alleles, including those containing ambiguous breakpoint regions. If genomic coordinates are given, VarSCAT has a function to carry out custom annotations using user provided BED files and can output the subregion of the wildtype and mutated DNA sequences that contain variants as well as the complementary sequence of the mutated sequence. With the tandem repeat annotation module, VarSCAT can annotate the local sequence contexts surrounding variants and putative tandem repeat regions with default or user-defined parameters for purity, composition, and size of putative tandem repeats.

To assess our newly developed VarSCAT tool, we used variants based on human reference assembly GRCh38 from the ClinVar database [44], Platinum Genome [45], the National Institute of Standards and Technology’s Genome in a Bottle (GIAB) [46,47], and the 1000 Genomes Project [48,49] to study the breakpoint ambiguities of indels and small variants (1–50 bp) in STR regions. Due to the varying algorithmic parameters used in different studies for STR detection, such as the minimum length of an STR and the tolerance of mismatches and indels between STR units, the definitions of an STR may vary widely and lead to highly variable interpretations [50–52]. In our study, we restricted our analysis to perfect STRs (except benchmarking of VarSCAT in the following section) with motif sizes of 1–6 bp based the common definitions in the literature [39,53–55]. We set the minimum length of an STR region to 10 bp based on a computational and experimental study into DNA polymerase-mediated strand slippage rates [56]. We set the minimum copy number to 10 for mononucleotide STRs, 5 for dinucleotide STRs, and 4 for tri- to hexanucleotide STRs, according to a previous study [56], a meta-analysis [57], and an *in silico* study into microsatellite distributions in the human genome [58] (S1 File: Sections S1–S3).

### Benchmarking VarSCAT against other methods for annotating variants in STR regions

We first analyzed annotations of variants in STR regions. High-confidence small variants located in the human reference genome GRCh38 chromosome 1 of two human individuals (HG002 and HG006) from the GIAB Consortium were selected. The current strategies for annotating variants in STR regions are as follows: 1) directly annotate variants in STR regions with a reference genome, 2) download ready-made STR annotations and then use available annotation tools to annotate variants, or 3) detect STR regions in a reference genome and then use the annotation tools to annotate variants. To incorporate various annotation strategies into benchmarking, we selected GATK (v4.1.9.0, ‘TandemRepeat’ function) [59] for direct annotation, TRF [32] and RepeatMasker [33] from UCSC Genome Browser’s ‘Simple Repeats’ and ‘RepeatMasker’ tracks (download date 11 February 2022) with additional filtration (S1 File: Section S2) to represent the ready-made STR annotations of a reference genome, and Krait (v1.3.3) [35] for detecting and annotating STRs against a reference genome. For annotations of the reference sequence GRCh38 chromosome 1, we used ANNOVAR (version: ‘$Date: 2019-10-24’) [36] as the annotation tool to annotate variants of GIAB HG002 and HG006 using VCF files. After collecting variants located in STR regions using different annotation methods, we used UpSet plots to view the overlaps among the different annotation sets (S1 File: Section S2).

**Fig 3:**
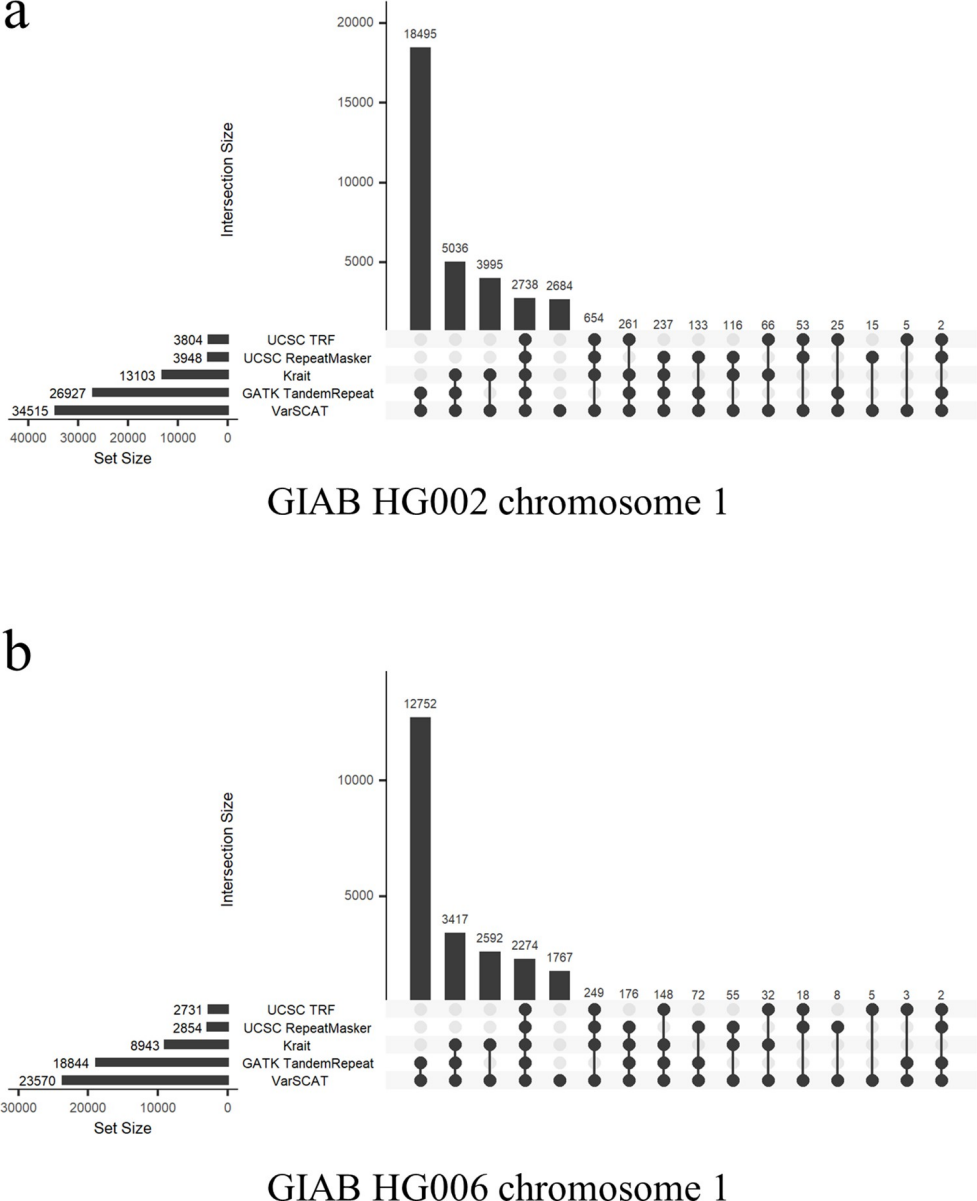
The benchmarking results of the VarSCAT tandem repeat annotation module. Benchmarking was performed for annotating small variants in perfect STR regions in chromosome 1 of **(a)** GIAB HG002, and **(b)** GIAB HG006. GATK ‘TandemRepeat’ function is an annotation method that directly takes a VCF file as the input; TRF and RepeatMasker from the UCSC Genome Browser’s ‘Simple Repeats’ and ‘RepeatMasker’ tracks represent a ready-made STR annotation approach; Krait is an annotation method for detecting perfect STRs with a reference genome.

Our results demonstrated that VarSCAT could annotate the largest collection of variants located in perfect STR regions than the other annotation methods tested (Fig 3). VarSCAT and GATK ‘TandemRepeat’ shared a large proportion of annotated variants because these two methods prefer to consider STRs with a short sequence context around variants instead of seeking larger tandem repeat regions with a longer sequence context. VarSCAT made some unique annotations because it not only considered the single reported position of a variant (‘POS’ column in the VCF file) but also considered the breakpoint ambiguity of a variant. Thus, VarSCAT can also annotate variants located partially in STR regions and variants directly adjacent to STR regions, whereas annotation tools like ANNOVAR only consider the single positions of variants when annotating VCF files with sequence annotations.

To test imperfect STR annotation, we loosened the STR criteria to STR regions which had at least 90% matches for their repeat motifs (S1 File: Section S1). Similar to the annotation of variants in perfect STR regions, our results showed that VarSCAT annotated a large collection of variants (S1 Fig). Since VarSCAT was able to annotate imperfect STRs unlike GATK ‘TandemRepeat’, more unique annotations were made by VarSCAT, while still sharing a large proportion of annotated variants with GATK ‘TandemRepeat’. Because of different definitions of an STR between tools, for imperfect STR annotation, other tools and methods annotated a small number of variants in STR regions that were not annotated by VarSCAT. We further used the whole variant set of GIAB HG002 to compare variant annotations of perfect and imperfect STR regions (90% matches) by VarSCAT. The results showed VarSCAT annotations for variants in imperfect STR regions led to a greater number of annotations, longer lengths, and higher copy numbers of STR regions than perfect STR regions annotated by VarSCAT (S2 Fig).

### Benchmarking VarSCAT for annotating indels with breakpoint ambiguity

**Fig 4:**
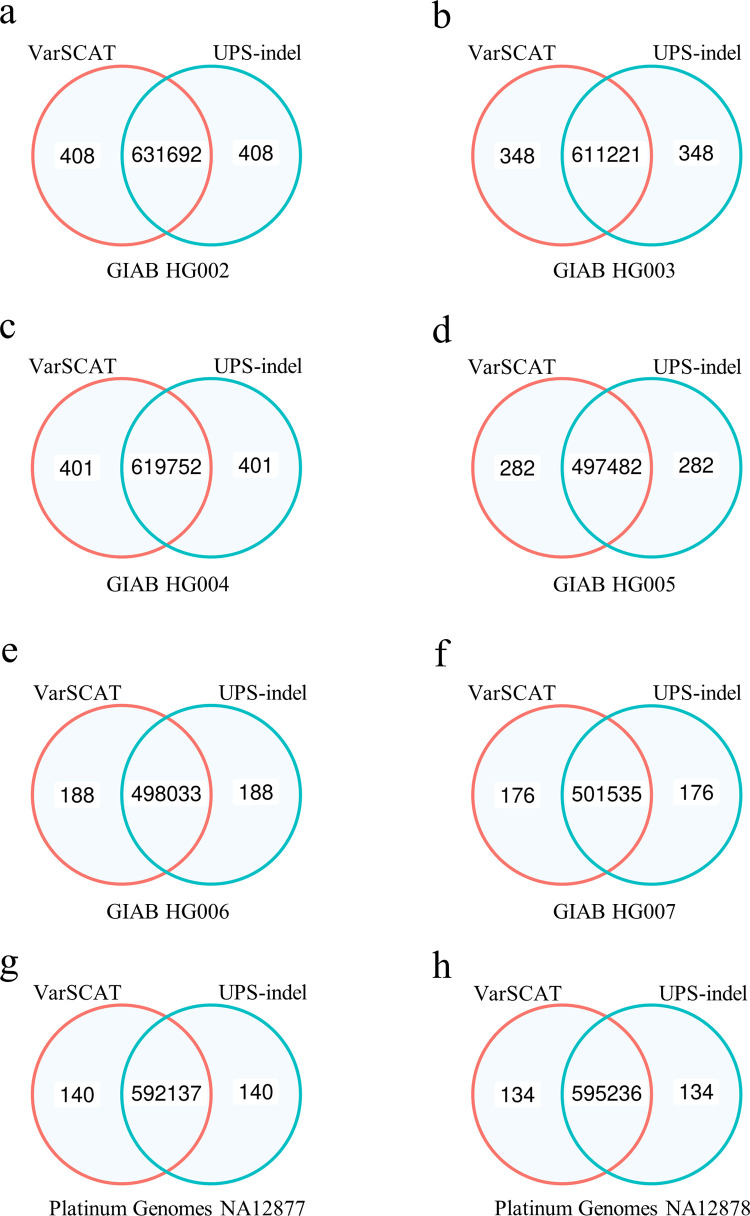
The benchmarking results of VarSCAT and UPS-indel for annotating indels with breakpoint ambiguity. Benchmarking was performed with indels of **(a)** GIAB HG002, **(b)** GIAB HG003, **(c)** GIAB HG004, **(d)** GIAB HG005, **(e)** GIAB HG006, **(f)** GIAB HG007, **(g)** Platinum Genomes NA12877, and **(h)** Platinum Genomes NA12878. Venn Diagrams were used to show the concordance of indel annotations between the tools. The numbers are the counts of indels annotated by each tool.

Besides annotating variants in STR regions, we then compared VarSCAT with UPS-indel for annotating indels with breakpoint ambiguity. Indels from eight human individuals were considered (GIAB HG002-HG007, Platinum Genomes NA12877, and NA12878). We compared the concordance between VarSCAT and UPS-indel for annotating 5’- and 3’-aligned positions of indels (S1 File: Section S2). The results demonstrated that the breakpoint ambiguity annotations of indels by VarSCAT and UPS-indel showed great consensus with small fractions of discordance (Fig 4). We further manually investigated these discordant indel annotations and found that UPS-indel failed to annotate unnormalized indels or complex indels correctly (S1 and S2 Tables, demonstrated from Platinum Genomes NA12878). For example, an unnormalized indel with a reference allele CATTC and an alternative allele CATC, or a complex indel with a reference allele CATTC and an alternative allele G can be correctly annotated by VarSCAT but not UPS-indel. The running time and maximum memory usage of VarSCAT and UPS-indel showed that VarSCAT can make variant annotations with shorter time and less memory than UPS-indel (S3 Table).

### Large proportion of human indels are breakpoint-ambiguous or located in duplicates

**Fig 5:**
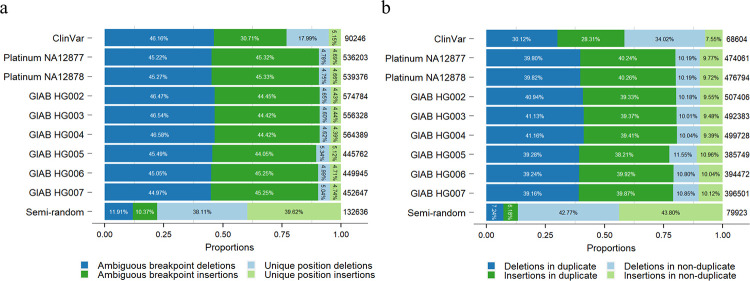
Proportions of ambiguous breakpoint indels and indels located in duplicates. The analysis was performed with indels from the ClinVar database, eight high-confidence human individual germline small variant sets, and one semi-random indel set. The proportions of deletions and insertions in different categories are shown separately: **(a)** the proportion of ambiguous breakpoint indels and **(b)** the proportion of indels in duplicate. The numbers on the right of each bar are the numbers of ambiguous breakpoint indels and indels in duplicate for each indel set, respectively.

To further study the proportions of ambiguous breakpoint indels in the human genome, we considered indels from the ClinVar database as clinically related indels and took the Platinum Genome NA12878 and NA12877 and GIAB HG002–HG007 indels as neutral germline indels (Fig 5 and S1 File: Sections S3 and S4). We defined an indel as ‘an ambiguous breakpoint indel’ if its 5’- and 3’- aligned positions differed. Our results showed that the majority of indels in the human genome had breakpoint ambiguities (Fig 5a). For the ClinVar database, more than 75% of all indels had breakpoint ambiguities, with 46% and 31% breakpoint-ambiguous deletions and insertions, respectively. For small germline indels in individual human samples, around 90% of all indels had breakpoint ambiguities, with breakpoint-ambiguous deletions and insertions around 45%.

We further restricted our criteria to analyze the proportion of indels located in duplicates. We defined ‘an indel located in a duplicate’ as one where a deletion occurred in a duplicated (or higher order) sequence, or an insertion either generated a novel duplication or extended an existing one. Our results showed that most indels in the human genome were found in duplicates, including nearly 60% of indels in the ClinVar database and around 80% of indels in the small germline indel sets of the eight human individuals (Fig 5b).

Indels in the ClinVar database had lower proportions of both breakpoint-ambiguous indels and indels located in duplicate than individual human germline small indel sets. The possible reason for this is that the average size of indels in the ClinVar database (mean: 56 bp and median: 3 bp) was larger than the average size of human germline small indels (for example, mean: 3 bp and median: 1 bp for Platinum NA12878). The larger indels contained more sequence diversity, thus making the proportions of breakpoint-ambiguous indels and indels located in duplicates lower.

Finally, we created a semi-random small indel set to validate our results (S1 File: Section S4). The total count and size distribution of the semi-random indels were simulated based on the indel set of Platinum Genome NA12878, but with indels randomly inserted into the human reference genome GRCh38. The proportions of breakpoint-ambiguous indels and indels located in duplicate in the semi-random small indel set showed large differences compared to the real human individual germline small indel sets. Only around 22% and 13% of the semi-random small indels were breakpoint-ambiguous and located in duplicates, respectively. Thus, our results demonstrated that human indels appeared in a specific sequence context, and the breakpoint ambiguity issue could not be ignored.

### Characteristics of small variants in STR regions of the human genome

**Fig 6:**
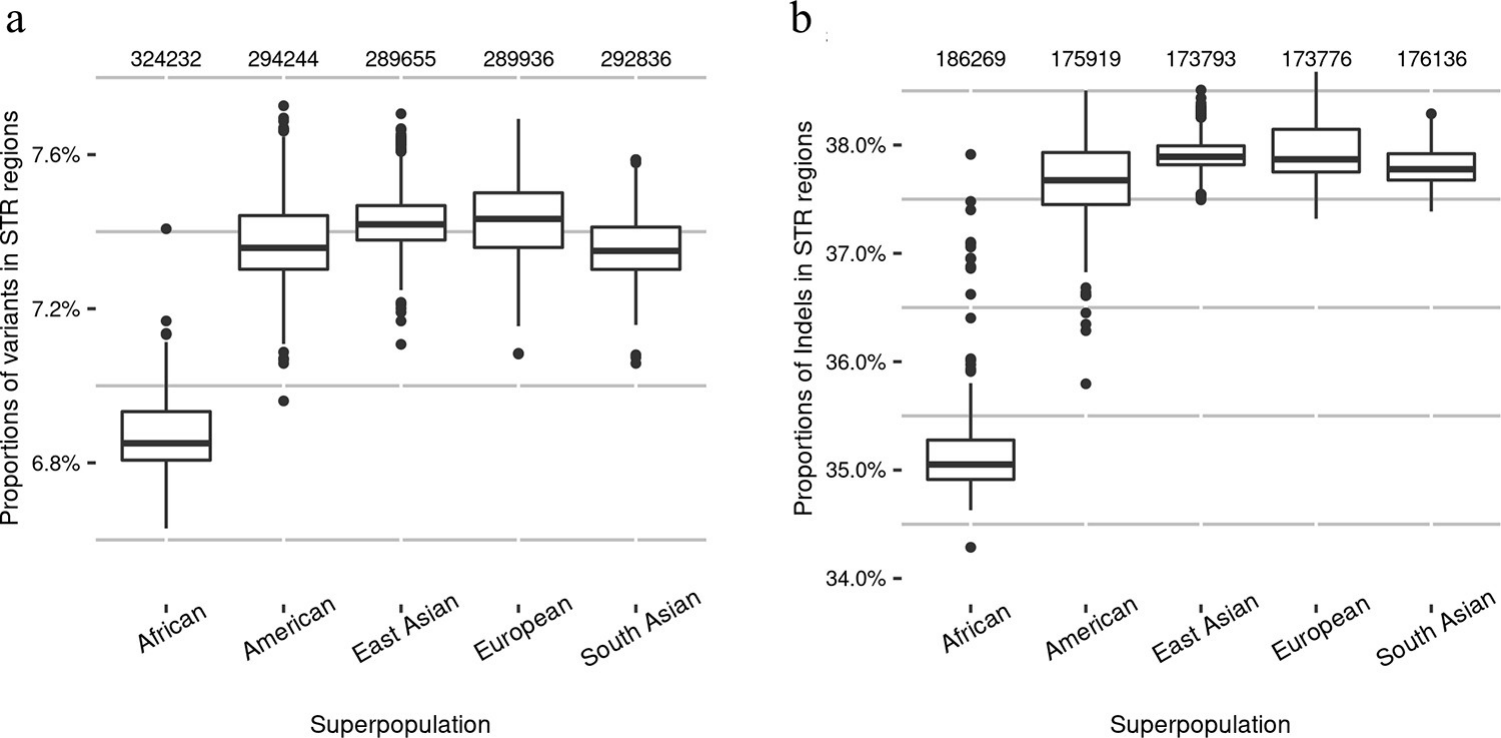
Proportions of small variants and indels in STR regions in different human superpopulations in the 1000 Genomes Project. (a) The proportions of small variants in STR regions and (b) the proportions of small indels in STR regions. The numbers at the top of each boxplot are the average numbers of variants or indels in the STR regions of each superpopulation. African: n = 671; American: n = 348; East Asian: n = 515; European: n = 522; South Asian: n = 492.

To study the proportions of variants in STR regions of the human genome in general, we analyzed small variants for 2,548 human individuals from the 1000 Genomes Project that were mapped against GRCh38 (S1 File: Sections S3 and S5). Our results showed that, on average, 7.3% of individual human germline small variants (including indels) were located in STR regions (Fig 6a). For individual human germline small indels, 37.1% of them were located in STR regions (Fig 6b). At the superpopulation level, the American, East Asian, European, and South Asian populations had similar average proportions of small variants and indels in STR regions, while the African population had a smaller average proportion but a higher average number of small variants and indels in STR regions compared with the other superpopulations. Similar results were obtained when we analyzed proportions of small variants and indels in STR regions at the subpopulation level (S3 Fig).

**Fig 7:**
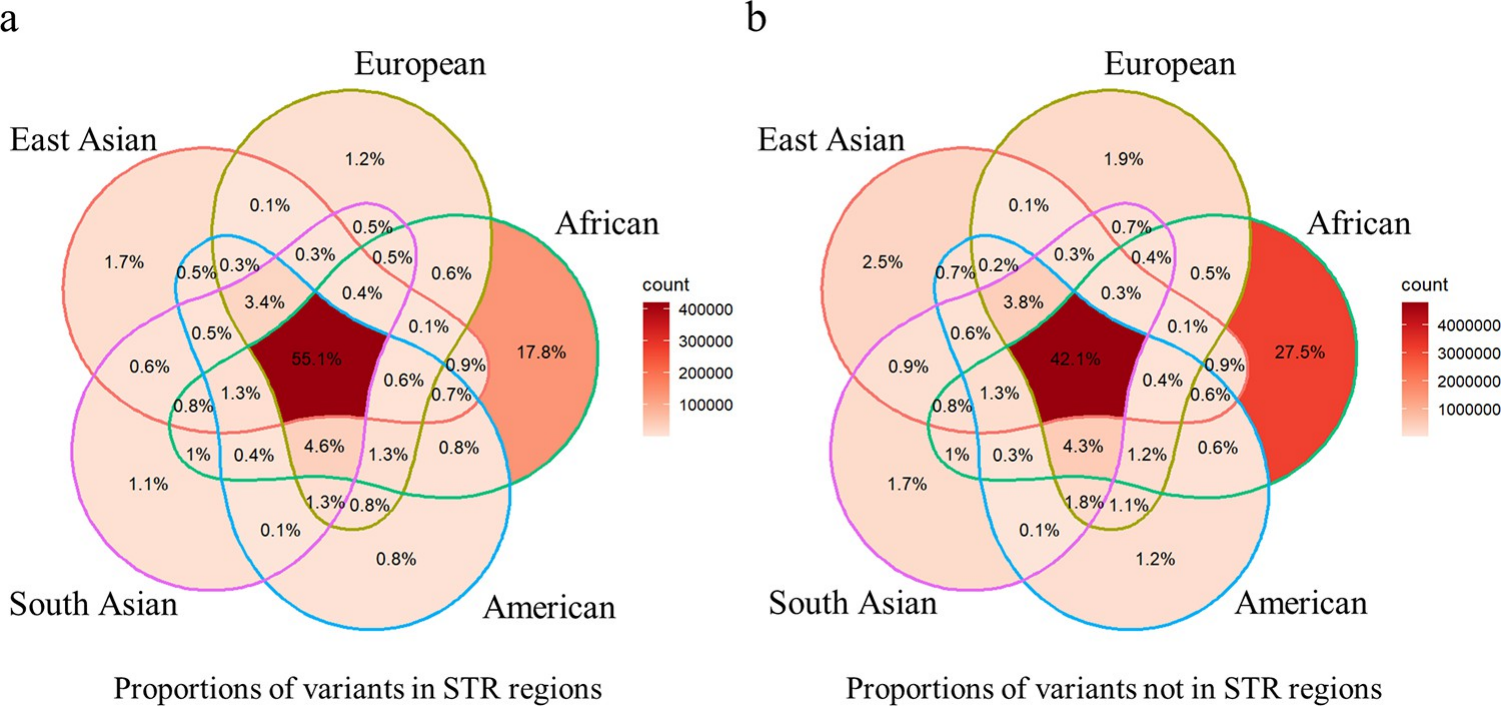
Proportions of small variants in STR and not in STR regions shared by human superpopulations in the 1000 Genomes Project. **(a)** The proportion of small variants in STR regions and **(b)** not in STR regions shared by superpopulations.

To further study the proportions of small variants in STR regions that were shared by the superpopulations, we chose common variants that had at least a 5% minor allele frequency among any of the superpopulations. The results showed that a large proportion (55.1%) of small variants in STR regions were shared by all five superpopulations, and that the proportion was higher than the proportion of small variants not in STR regions (42.1%; Fig 7). Furthermore, the proportions of small variants in STR regions that were superpopulation specific were smaller (0.8–17.8%) than the proportions of small variants not in STR regions (1.2–27.5%). This trend was seen in all five superpopulations. The results indicated that small variants in STR regions were more common and more often shared between superpopulations than small variants not in STR regions.

**Fig 8:**
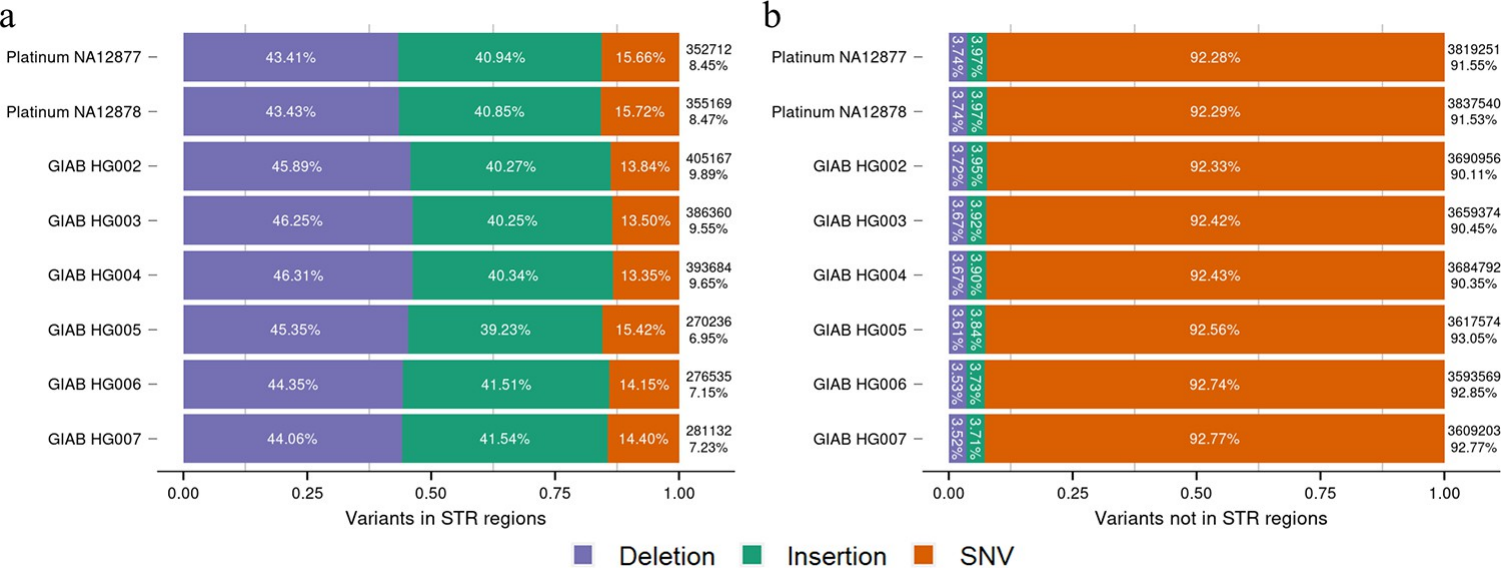
Proportions of different types of small variants in the STR and not in STR regions. **(a)** The proportions of deletions, insertions, and single nucleotide variants (SNVs) in STR regions and **(b)** not in STR regions. The analysis was performed on high-confidence small variant sets from two individuals from the Platinum Genome and six individuals from GIAB. The numbers on the right of each bar show the numbers of small variants in STR regions or not in STR regions for the individuals. The percentages on the right of each bar donate the proportions of small variants in STR regions or not in STR regions among all variants for the individuals.

To investigate the small variants in STR regions of single individuals, we analyzed high-confidence small variant sets of two samples from the Platinum Genome and six samples from GIAB (Fig 8 and S1 File: Sections S3 and S5). The results showed that among the small variants in the STR regions, the deletions had the largest proportion (around 45%), followed by insertions of (around 40%) and single nucleotide variants (SNVs, around 15%). In contrast, among the small variants not in STR regions, SNVs were the dominant variant type (above 92%), while both deletions and insertions had low proportions (around 4%).

We next selected small indels in STR regions that spanned only one STR region (on average, 94.2% of total small indels in STR regions in eight individuals) and studied the correlations between indel sizes and STR motif sizes. Our results showed that the majority of small indels in the STR regions were the same size as the STR motif itself (e.g., indels of 2 bp were located predominantly in the dinucleotide STR regions). Indel sizes were also enriched with multiples of STR motif sizes (e.g., dinucleotide STR regions contained indels affecting 2, 4, 6, etc. nucleotides). These findings applied equally to both deletions and insertions (S4–S9 Figs). As expected, the numbers of STR regions that contained small indels decreased when the STR copy numbers or STR motif sizes increased (S10–S15 Figs).

The overall proportion of small variants in the STR regions for the eight high-confidence human small variant sets was higher than for the individuals in the 1000 Genomes Project (Figs 6 and 8). This may be due to improved sequencing techniques and computational variant calling methods that detected high-confidence small variants from difficult regions of the human genome, including STR regions. While the 1000 Genomes Project generated variants with the Illumina NGS short-read platform, GIAB produced high-confidence variant sets with various sequencing platforms, including NGS short-read and third generation, long-read sequencing platforms, as well as variant calling methods based on deep learning approaches. Our results indicated that indels were the most common small variant type in STR regions, and the size distribution of small indels in STR regions had high correlations with the size of the STR motifs.

**Fig 9:**
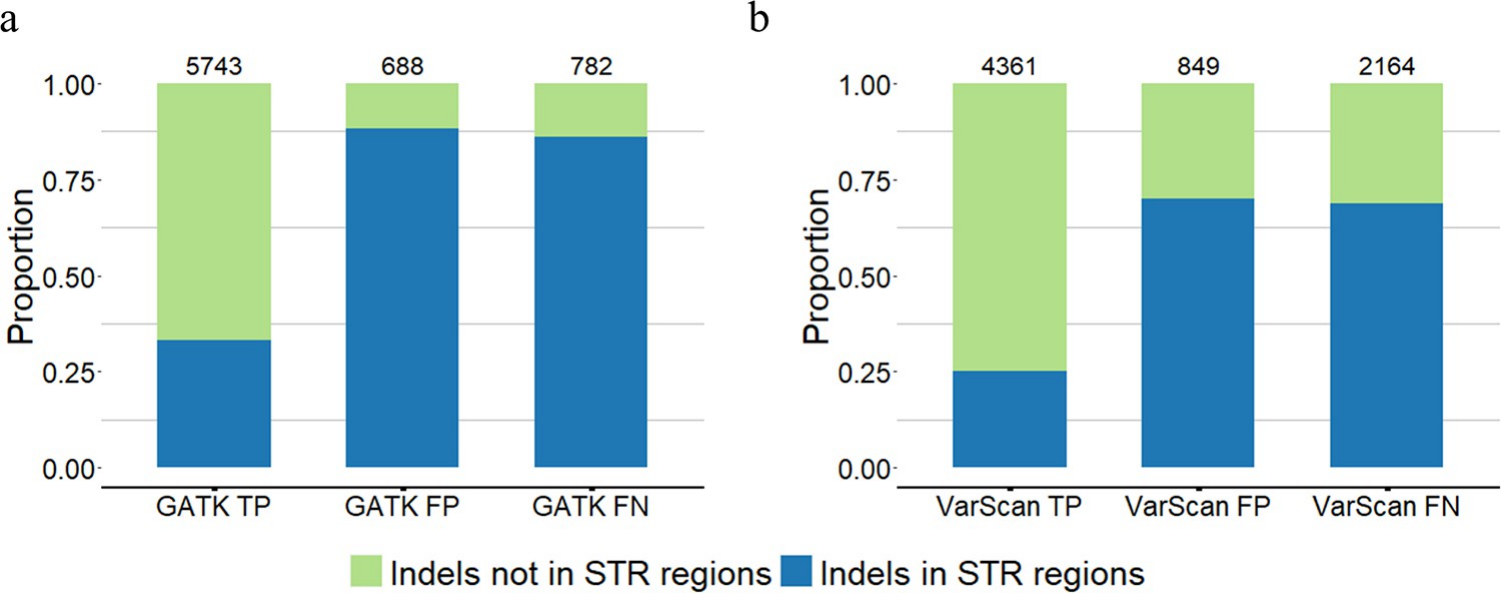
Indel calling results of GATK HaplotypeCaller and VarScan with the whole exome sequencing data of GIAB HG002. **(a)** The indel calling results of GATK HaplotypeCaller and **(b)** VarScan. The evaluation results of true positive (TP), false positive (FP), and false negative (FN) of indel calls were generated using hap.py. The numbers on top of each bar show the total number of indels in this category. The indels in STR regions and not in STR regions are marked with different colors.

We further assessed how STR regions can affect indel calling. For that, we used the whole exome sequencing indel calling results of GATK HaplotypeCaller (v4.0.1.2) and VarScan (v2.4.3) for GIAB HG002 from our previous study [27]. The indel calling results were evaluated with GIAB HG002 v4.2.1 GRCh37 using hap.py [60] (S1 File: Section S5). In general, GATK HaplotypeCaller performed better than VarScan for calling small indels (Fig 9). Regardless of the tool, the majority of the true positive indel calls were not located in STR regions, whereas the majority of false positive and false negative indel calls were located in STR regions. These results suggest that STR regions might be main difficulty for indel calling (Fig 9).

## Discussion

The sequence contexts can substantially influence genomic variants. Assessing the sequence contexts of variants can help in understanding potential technical issues, such as biologically equivalent indels caused by breakpoint ambiguity, as well as biological significances, such as variants in the STR regions. To learn the sequence contexts of variants using sequencing data, annotation tools such as ANNOVAR can be integrated with other annotation sources to build custom pipelines for annotating variants. However, building such pipelines requires great effort, which can be time-consuming and difficult. Hence, we developed a single command-line-based computational tool VarSCAT, which takes a VCF file and a reference sequence FASTA file as inputs. VarSCAT has various functions for annotating breakpoint ambiguities, flanking bases of variants, HGVS nomenclature, distances between adjacent variants, tandem repeat regions, and custom annotations of genomic variants, as well as for extracting wildtype and mutated DNA sequences by considering all the variants within a user-defined region. The VarSCAT results provide users with valuable information about the sequence contexts of variants, which can be used for purposes such as variant calling result filtration and variant nomenclature.

To demonstrate the utility of VarSCAT, we used its flexibility to analyze high-confidence human germline small variant sets from Platinum Genome, GIAB, and the 1000 Genomes Project, and clinically related indels from the ClinVar database to study the sequence contexts of variants. Our benchmarking results for the annotation of variants in STR regions showed substantial discordance between the different methods and strategies, indicating that the current methods and strategies may underestimate the proportions of variants in STR regions. Some STRs that were not annotated by other methods in our benchmarking may be because of the strict underlying criteria of other methods that filter them out or because they were part of large tandem repeats and were thus ignored by other methods. Our results showed that the majority of human indels had breakpoint ambiguities, consistent with a previous study [23]. Although STRs occupy only about 3% of the human genome [9], our study showed that on average, more than 7% of human germline small variants were in STR regions—especially small indels, which were the most dominant variant type in STR regions. Small variants in STR regions were more common than these not in STR regions across human superpopulations, and the sizes of small indels in STR regions correlated highly with the sizes of the STR motifs. Our results agreed with previous research indicating that DNA strand slippage is the main molecular mechanism underpinning indel generation [61], and that the current African populations have larger genetic variations than the non-African populations [13].

Although VarSCAT can annotate variant sequence contexts comprehensively in a high-throughput manner, our study and results still have some limitations. Due to the inconsistent criteria for imperfect STRs, such as mismatches and indels tolerance, we limited our research to perfect STRs, which may have led to an underestimation of the proportions of variants in STR regions. STR regions annotated by VarSCAT may not always have biological significance, and further validations are needed, such as studies of the functions of STRs and variants. The current variant annotation methods only consider the single reported positions of variants from VCF files (“POS” column). However, some variants, especially indels, are not only affect a single position but also a region on genome. Thus, the current methods may miss annotations if there is only a partial overlap between variants and STR regions. Although VarSCAT can annotate variants that only partially overlap or directly adjacent to an STR, we focused on high-confidence individual human small variant sets in our analysis. The high-confidence small variant sets had high precision and covered much of the human genome, but they still excluded some difficult regions that were enriched with STRs and limited the sizes of variants due to difficulties in variant genotyping [45–47].

The data we used from the 1000 Genomes Project were the most recent variant call sets, based on the current human reference genome assembly GRCh38. The data contained 2,548 samples, which gave us an adequate sample size to study the general proportions of small variants in the STR regions of human individuals. However, the data contained only biallelic variants. Although the majority of variants in Phase Three of the 1000 Genomes Project are biallelic [49], we still may have systematically underestimated the proportions of small variants in STR regions because these variants are often highly multiallelic [62]. With improved sequencing techniques, such as long-read sequencing and corresponding variant calling methods, the STR-enriched regions, such as centromeres and telomeres, could be precisely sequenced, and the variations among individuals could be genotyped accordingly. These improvements have the potential to further improve studies of variants in STR regions using VarSCAT and bring more insights into genetic mechanism among human diseases, genomic variants, and STR regions.

Besides, VarSCAT itself also has some limitations. First, the current version of VarSCAT lacks ability to predict or distinguish whether variants called by variant calling tools are true positives or false positives. The false positive variant calling results may not only depend on the sequence context of genomic variants. Other factors, such as library preparation, sequencing platform, or sample quality, also play important roles in accurate variant calling. To consider all these factors, the further development of VarSCAT could utilize machine learning methods, trained with variants from different platforms, quality standards, or other features, to predict likely false positive variants. Additionally, the current version of VarSCAT annotates variants from a pre-called variant set. However, the variant calling tools may not be able to call all variants in STR regions. The future development of VarSCAT could directly utilize sequencing data to detect variants in STR regions instead of only annotating pre-called variants.

## Methods and materials

### VarSCAT annotation modules

VarSCAT is a computational tool which takes a VCF file and a reference sequence as inputs to annotate the sequence contexts of genomic variants. First, all the input variants are processed by the variant normalization module to splits any multiallelic variants into biallelic variants and then normalizes them as parsimonious and left aligned (for definitions see the next paragraph). The adjacent sequence annotation module annotates variants related to breakpoint ambiguities and the tandem repeat annotation module can annotate putative tandem repeat regions that contain variants. These two modules and their functions can be used together or separately. The design of each module is described as follows.

### Variant normalization module

For VarSCAT’s first analytical step, we built a module that normalizes input variants parsimonious and left aligned after separating any potential multiallelic variants into biallelic variants. A variant is parsimonious if (and only if) it is represented in as few nucleotides as possible without an allele of zero length [63]. A variant is left aligned if (and only if) it is no longer possible to shift its position to the left while keeping the length of all its alleles constant [63]. This variant normalization module can be used to analyze input variants in a standardized way, without further concerns about multiple representations of a variant.

VarSCAT requires a VCF input file. The variant information for chromosomes (‘CHROM’ column), positions (‘POS’ column), reference alleles (‘REF’ column), alternative alleles (‘ALT’ column), identifiers (‘ID’ column) and genotypes (‘GT’ section in the ‘INFO’ column) are read by the PyVCF package [64]. The variant normalization algorithm is similar to the vt tool [63]. For normalization, VarSCAT extracts indels and multi-nucleotide variants (MNVs) of which REF or ALT alleles with length > 1 bp. SNVs have single positions with single nucleotide substitutes and do not need to be normalized. First, for indels and MNVs, if the rightmost sequence nucleotides of REF and ALT are the same, VarSCAT trims this common last nucleotide until the uncommon nucleotides are met. During this trimming, if the sequence length of any one of REF or ALT is zero, then both REF and ALT are extended by one nucleotide to the left based on the reference genome sequence, and the position of the variant is updated. After trimming to the rightmost sequence nucleotides, VarSCAT examines the leftmost nucleotides of the variants. If both REF and ALT lengths are longer than one and have common nucleotides, then the tool trims the leftmost nucleotides of REF and ALT and updates the position of the variant. After the normalization process, the chromosomes, positions, REFs, ALTs, IDs, and genotypes of all the input variants are stored.

### Adjacent sequence annotation module

With the adjacent sequence annotation module, VarSCAT returns each variant record as a row in a text file. Each record from this module contains basic variant information (chromosome, position, REF, ALT, ID, and genotype) and optional information, including 5’-aligned position, 3’-aligned position, 3’ edge position (the rightmost base position of a variant), the flanking bases of REF and ALT, the sequence variant nomenclature recommended by HGVS (version: 20.05) [24], and the distance to the next variant on 3’ direction by activating additional parameters. Furthermore, if the user provides a genomic region, VarSCAT can output the region-specific wildtype and mutated DNA sequence, and its reverse complement sequence in a FASTA file. Besides, VarSCAT also has abilities to annotate variants with user provided, custom annotation files in BED format for specific genomic regions.

Because a point nucleotide change occurs only at a fixed position, there is no breakpoint ambiguity for SNV, MNV, and complex indel (for example, REF: CATTC, ALT: G). For the variants that contain a point nucleotide change, the 5’-aligned and 3’-aligned positions are the positions of the first nucleotide of the variant (the same as the position of the variant), and the 3’ edge position is the position of the last nucleotide of the variant. For indels, we first compute the 3’ edge positions. VarSCAT first extracts the indel pattern, which is the leftmost nucleotide trimmed sequence of REF or ALT for deletion and insertion, respectively. If the pattern of an indel with length *n* is a nucleotide subsequence (*a*_*1*_, *a*_*2*_, *a*_*3*,_ …, *a*_*n*_), the adjacent reference sequence in the 3’ direction to the indel is a nucleotide sequence (*r*_*1*_, *r*_*2*_, *r*_*3*,_ …) with genomic coordinates (*c*_*1*_, *c*_*2*_, *c*_*3*,_ …), so the initial 3’ edge position of the indel is the genomic coordinate *c*_*1*_-1. VarSCAT checks whether the leftmost nucleotide of the indel pattern *a*_*1*_ is the same nucleotide as the first 3’-direction reference sequence nucleotide *r*_*1*_. If *a*_*1*_ is the same nucleotide as *r*_*1*_, then the indel pattern permutes into (*a*_*2*_, *a*_*3*_…*a*_*n*_, *a*_*1*_) and the 3’ edge position updates to the genomic coordinate *c*_*2*_-1. In the next round, the updated leftmost nucleotide of the indel pattern *a*_*2*_ is compared with the second 3’ direction reference sequence nucleotide *r*_*2*_. If they are the same, VarSCAT continues the variant pattern permutation, and the 3’ edge position is updated; if they are not the same, VarSCAT terminates the process and reports the 3’ edge position. For deletion, the 5’-aligned position is the position of the leftmost possible nucleotide of the deleted sequence on the reference coordinate, and the 3’-aligned position is the position of the rightmost possible nucleotide of the deleted sequence on the reference coordinate (3’ edge position) minus the length of the deletion pattern; for insertion, the 5’-aligned position is the position of the leftmost possible 5’ direction adjacent nucleotide of the inserted sequence on the reference coordinate (the same as the position of variant), the 3’-aligned and 3’ edge positions are the same as the position of the rightmost possible 5’ direction adjacent nucleotide of the inserted sequence on reference coordinate. With the 5’-aligned and 3’ edge positions, the 3’ direction distance to the next variant from the input VCF can be calculated.

We computed the HGVS nomenclature of variants followed by the HGVS recommendations (detailed in https://varnomen.hgvs.org/recommendations/DNA/) with a custom algorithm and script using the 5’-aligned and 3’-aligned positions. Also, if the ambiguity region of a variant is known, VarSCAT can output flanking bases of the variant, including the potential ambiguity sequences of REF and ALT. If the user provides a genomic region, VarSCAT can extract a wildtype DNA sequence from this region together with variants within this region to create the mutated DNA sequence. The reverse complement sequence of a mutated DNA sequence was created using the Biopython package [65]. Besides, VarSCAT also has abilities to annotate variants with user provided, custom annotation files in BED format for specific genomic regions using pybedtools package [66].

### Tandem repeat annotation module

VarSCAT uses a local sequence context comparison algorithm designed according to the idea of tandem repeat definition to explore the tandem repeat regions that either overlap a variant or are adjacent to a variant.

#### Initiation of a variant site

Let *min_unit* be the minimum size of a conserved repeat motif, and *max_unit* be the maximum size of a conserved repeat motif, defined by the user or by default. For each input variant that does not contain ‘N’, VarSCAT first defines the variant site based on the 5’-aligned and 3’ edge position of a variant from the adjacent sequence annotation module. The variant site is the coordinates between two nucleotides on the reference sequence, which located in the 5’ and 3’ directions adjacent to the 5’-aligned and 3’ edge positions of the variant, respectively. For a known variant site, VarSCAT creates a window size set *W* = {*w*_*1*_, *w*_*2*_, *w*_*3*,_ …} in which *w*_*i*_ is a certain window size and *W* contains window sizes with a range of *min_unit* ≤ *w*_*i*_ ≤ *max_unit*. For each certain window size *w*_*i*_, VarSCAT creates a set of nucleotide subsequences *R* = {*r*_*1*_, *r*_*2*_, *r*_*3*,_ …}, in which *r*_*i*_ is subsequence with size *w*_*i*_ as a candidate conserved repeat motif and *R* contains all the possible subsequences with at least one base pair overlapped or directly adjacent to the variant site in the 5’ to 3’ direction on the reference sequence.

#### The local sequence context comparison algorithm for repeat units searching

If *r*_*i*_ with size *w*_*i*_ is one candidate conserved repeat motif. VarSCAT first takes the 5’ direction to search for potential repeat units. The algorithm creates a set of nucleotide subsequences *S* = {*s*_*0*_, *s*_*1*_, *s*_*2*,_ …, *s*_*wi*_}. Nucleotide subsequences in *S* have the same size of *w*_*i*_ and each *s*_*i*_ is a nucleotide subsequence created by moving window *w*_*i*_ 1 bp away from *r*_*i*_ in the 5’ direction per moving step on the reference sequence. Each moving step can form a gap, and the gap distance between *r*_*i*_ and *s*_*i*_ is from 0 bp to a maximum of *w*_*i*_ bp or a user-defined fixed maximum gap tolerated distance. Each *s*_*i*_ is treated as a potential repeat unit and set *S* ensures that VarSCAT captures all subsequences as potential repeat units within the maximum gap-tolerated distance. With all possible *s*_*i*_ in *S*, a similarity comparison between *r*_*i*_ and *s*_*i*_ is performed. Due to the same string lengths between *r*_*i*_ and *s*_*i*_, the number of common bases at each string location is used as the similarity comparison score. For all possible *s*_*i*_ and their corresponding similarity comparison scores, the *s*_*i*_ with the highest score is chosen, and a corresponding gap distance is determined (if there are equal best scores, VarSCAT will choose the *s*_*i*_ with the smallest gap distance). If *s*_*i*_ with the highest similarity comparison score passes the default or user-defined similarity threshold with *r*_*i*_, *s*_*i*_ is treated as one repeat unit, and the gap distance and the number of mismatched nucleotides between *r*_*i*_ and *s*_*i*_ are stored before the next round is executed. For the next round, a new set of subsequences *S* is created in the 5’ direction of *s*_*i*_ and the same local sequence context comparison and criteria are applied to search the next repeat unit. The process is terminated when all the possible elements of *S* in the *n*th round fail to pass the similarity threshold with *r*_*i*_, and the same process is performed but in the 3’ direction from *r*_*i*._ After searching and collecting all possible repeat units in both the 5’ and 3’ directions, a candidate tandem repeat region of motif *r*_*i*_ is formed, and the numbers of matched nucleotides, mismatched nucleotides, gaps, and the copy number in this tandem repeat region are stored.

The VarSCAT results had similar underpinning technical concepts, such as ‘Match’, ‘Mismatch’, and ‘Gap’ to a global pairwise alignment based on the Needleman–Wunsch algorithm [67]. The VarSCAT results can be seen in terms of a Needleman–Wunsch algorithm that aligns the VarSCAT-defined tandem repeat region of motif *r*_*i*_ with an ideal perfect tandem repeat region (no mismatches or gaps) of the same motif *r*_*i*_ under the same copy number. If the copy number of the candidate tandem repeat region is larger than the default or user-defined minimum copy number, the alignment score (Eq 1) is calculated for the candidate tandem repeat region with the default or user defined match score (MS), mismatch score (MIS), and gap score (GS). We defined a repeat score as the alignment score divided by the length of the candidate tandem repeat region and then multiplied by the corresponding copy number (Eq 2). The conserved repeat motif *r*_*i*_, copy number, size of the conserved repeat pattern, start and end positions of the tandem repeat region, repeat score, alignment score, match percentage, mismatch percentage, gap percentage, and history records from every local sequence context comparison round for the start and end positions, repeat patterns, number of mismatches, and gaps of all the candidate tandem repeat regions are stored for post-processing quality control. Due to DNA having four different types of bases (A, T, G and C), during the first four rounds, there may be no consensus nucleotide pattern as a potential motif for local sequence context comparison. Therefore, during the first four rounds, *r*_*i*_ is the subsequence that created for the variant site. After the fifth round (if applicable), *r*_*i*_ is updated to the consensus sequence motif (*r*_*i*_, *s*_*i*_, *s*_*i2*_, *s*_*i3*,_ …, *s*_*in*_). Every round after the fifth round uses the updating consensus sequence motif for the local sequence context comparison, and the alignment results of the first four rounds are replaced with the conserved sequence motif.


AlignmentScore=MS×matchbases+MIS×mismatchbases+GS×gapbases (1)

$$Repeat\;Score=\frac{Alignment\;Score}{length\;of\;the\;TR\;region}\times copy\;number$$ (2)

#### Post-processing quality control

Post-processing quality control is applied using the default or user-defined parameters of 1) the minimum alignment score to control the minimum size of a tandem repeat region and 2) the minimum match percentage to control the repeat purity of a tandem repeat region. A tandem repeat region for a variant is stored if the tandem repeat region passes the quality threshold. If a variant has at least one candidate tandem repeat region after searching but no tandem repeat passes the quality threshold, a trimming algorithm is applied to the tandem repeat region with the best alignment score. We assumed that the 5’ and 3’ tails of a tandem repeat region would share less similarity and contain more gaps between the conserved repeat pattern, which could cause the alignment score and the match percentage to decrease below the quality threshold. VarSCAT trims the historical records of the repeat units by searching the results for both the 5’ and 3’ tails of a candidate tandem repeat region until the copy number is less than the threshold. If, during the trimming process, the match percentage and alignment score exceed the quality threshold, the trimming process is terminated, and the corresponding values of the candidate tandem repeat region are recalculated. Any candidate tandem repeat region of a variant site that meets the criteria is stored with its conserved repeat motif, copy number, repeat score, alignment score, percentage of matches, percentage of mismatches, percentage of gaps, and repeat region positions for further analysis.

#### Remove redundancy

After all potential candidate tandem repeat regions of a variant site are discovered, it is still possible for redundant tandem repeat regions to be recorded. The conserved repeat motifs of a redundant repeat region are usually multiples of each other, but only the most concise conserved repeat motif is needed. VarSCAT first ranks all the candidate tandem repeat regions with repeat scores in descending order, favoring the tandem repeat with the highest copy number. Starting with the highest repeat score candidate tandem repeat, all candidate tandem repeats are compared in a pairwise manner. We defined a redundancy ratio to filter out redundant tandem repeat regions. The redundancy ratio is the ratio of the size of overlapped regions and the maximum covered regions of the two pairwise compared tandem repeats on reference sequence. If the redundancy ratio is > 0.5, meaning that the size of an overlapped region is larger than half of the maximum covered region of two tandem repeats, VarSCAT considers one of the two tandem repeats as a redundant tandem repeat. For each tandem repeat in pairwise comparison, VarSCAT calculates the nucleotide frequency difference between its conserved repeat motif and the whole tandem repeat region. The tandem repeat with the smallest nucleotide frequency difference remains, and the other tandem repeat is dropped. The GC% and changes of copy numbers caused by variants are calculated for all remaining tandem repeat regions, which are reported as the final tandem repeat regions for the variant.

### Data sets

We investigated several high-confidence genomic variant data sets that either included variants with clinical significance or represented the natural distribution of human germline variants. We selected variants from the ClinVar database [44], two high-confidence small variant calls for human individuals NA12877 and NA12878 from Platinum Genomes [45], six high-confidence small variant calls (v4.2.1) for human individuals HG002-HG007 from the GIAB Consortium [46,47], and the integrated phased biallelic variant sets on GRCh38 for 2,548 human individuals from the 1000 Genomes Project [48,49]. An extended description of the data selection and processing is provided in S1 File: Sections S3–S5.

### ClinVar database

The ClinVar database (data archiving date: 2022/01/09) is a public archive hosted by the National Center for Biotechnology Information, which holds the relationships among human genome variations and phenotypes with supporting clinical evidence. The records in this database are alleles that have been mapped to reference sequences and reported according to the HGVS nomenclature standard, together with their clinical significance or supporting evidence about the effects of the variations [44]. The indels on chromosomes 1–22, sex chromosomes, and the mitochondrial chromosome in the ClinVar database were selected for the indel analysis of breakpoint ambiguity and duplicate.

### Platinum Genome

Platinum Genome is a high-confidence human small variant set that contains two human individuals (NA12877 and NA12878) from the CEPH pedigree 1463. The high-confidence variant set was generated using PCR-free whole genome sequencing for four grandparents, two parents (NA12877 and NA12878), and 11 children, and the calling variants in each genome using several publicly available, highly accurate variant calling algorithms. Platinum Genome used haplotype transmission information for this pedigree to create a high-confidence variant set containing 4.7 million SNVs and 0.7 million small indels that ranged from 1–50 bp [45].

### GIAB

The GIAB high-confidence small variant sets contain seven human samples (NA12878 and two son/father/mother trios of Ashkenazi Jewish and Han Chinese ancestry). High-confidence variant sets were generated using an integration pipeline of computational tools with sequencing data from multiple technology platforms, including both short-read and long-read sequencing platforms. In our study, we used GIAB high-confidence variant sets v4.2.1 of the trios of Ashkenazi Jewish and Han Chinese ancestry (HG002–HG007), in which more SNVs and indels located in difficult-to-map regions were added and covered 92% of the autosomal GRCh38 assembly [46,47].

### The 1000 Genomes Project

The 1000 Genomes Project is a comprehensive catalogue of common human genetic variations with allele frequencies of at least 1% in the populations studied. The data were produced by applying whole-genome sequencing for the human reference genome GRCh38 [48] to a diverse set of individuals from multiple populations. In our study, we used a set of biallelic SNVs and indels from 2,548 samples of 26 populations that were generated by the 1000 Genomes Project based on the human reference genome GRCh38. The variant call set was produced using a multi-caller approach, which integrated the call sets before final genotyping and phasing [49].

### Benchmarking of VarSCAT with other tools

For STR region variant annotation, variants in VCF files for chromosome 1 of two human individuals (HG002 and HG006) from GIAB were selected after splitting multiallelic variants (GATK v4.1.9.0, ‘—split-multi-allelics’) and left alignment (vt v0.57712, ‘left-aligning’). The ‘Simple Repeat’ and ‘RepeatMasker’ tracks of chromosome 1 of human reference hg38, representing TRF and RepeatMasker, respectively, were downloaded from the UCSC genome browser (download date 11 February 2022) and filter for perfect STRs and imperfect STRs with at least 90% matches for their repeat motifs. The STR records generated by Krait (v1.3.3) were perfect STRs by the “SSRs” function and imperfect STRs by combining STRs of the “SSRs” and “iSSRs” functions. All these STR records were then filtered based on the minimum lengths, copy numbers, and match percentages of STRs, as mentioned in the ‘Results’ section. We used a custom R script to create BED files for these STR records and used ANNOVAR (version: ‘$Date: 2019-10-24’) to annotate variants in VCF format. The GATK ‘TandemRepeat’ (v4.1.9.0) annotation results which directly took VCF files as inputs, can only annotate perfect STRs. The STR regions annotated by GATK ‘TandemRepeat’ were further filtered according to our criteria of the minimum length and copy numbers. VarSCAT also directly took VCF files as inputs, and we set VarSCAT parameters to annotate variants in perfect STR regions according to our criteria of the minimum length and copy numbers in ‘Result” section’. For imperfect STR region annotations, imperfect STR regions should have at least 90% matches for their repeat motifs (details are given in S1 File: Sections S1 and S2).

For variants breakpoint ambiguity annotation, indels were selected using VCFtools (v0.1.17) [68] from eight high-confidence, human variant sets GIAB HG002-HG007, and Platinum Genomes NA12877 and NA12878. The eight indel sets were annotated by VarSCAT and UPS-indel. The concordance of indel breakpoint ambiguity annotations between VarSCAT and UPS-indel was performed by comparing 5’- and 3’-aligned positions of indels annotated by the two tools. Venn Diagrams were applied to show the concordance between annotations. The running times and maximum memory usages of VarSCAT and UPS-indel were measured with high performance computer clusters with Intel Xeon Gold 6230 CPU @ 2.10GHz (details are given in S1 File: Section S2).

## Supporting information

S1 Fig The benchmarking results of the VarSCAT tandem repeat annotation module with imperfect STRs. The benchmarking was performed with small variants of chromosome 1 of **(a)** GIAB HG002, and **(b)** GIAB HG006. The numbers are the counts of variants annotated by each tool.
(TIF)

S2 Fig The annotations of variants in perfect and imperfect STR regions (90%) by VarSCAT. **(a)** the barplots of numbers of annotated STR regions. Different colors indicated the different sizes of repeat motifs and the numbers on top of each bar were the total numbers of annotated STR regions, **(b)** the boxplots of length of annotated STR regions. The numbers in the middle of each box were the average lengths of annotated STR regions, and **(c)** the boxplots of copy number of annotated STR regions. The numbers in the middle of each box were the average copy numbers of annotated STR regions.
(TIF)

S3 Fig The proportions of small variants and indels in STR regions in different human subpopulations of the 1000 Genomes Project. **(a)** The proportions of small variants in STR regions and **(b)** the proportions of small indels in STR regions. ACB, African Caribbean in Barbados, GWD, Gambian in Western Division Mandinka, ESN, Esan in Nigeria, MSL, Mende in Sierra Leone, YRI, Yoruba in Ibadan Nigeria, LWK, Luhya in Webuye Kenya, ASW, People with African Ancestry in Southwest USA, PUR, Puerto Ricans in Puerto Rico, CLM, Colombians in Medellin Colombia, PEL, Peruvians in Lima Peru, MXL, People with Mexican Ancestry in Los Angeles CA USA, CHS, Southern Han Chinese, CDX, Chinese Dai in Xishuangbanna China, KHV, Kinh in Ho Chi Minh City Vietnam, CHB, Han Chinese in Beijing, China, JPT, Japanese in Tokyo Japan, GBR, British in England and Scotland, FIN, Finnish in Finland, IBS, Iberian Populations in Spain, CEU, Utah residents with Northern and Western European ancestry, TSI, Tuscans in Italy, PJL, Punjabis in Lahore Pakistan, BEB, Bengalis in Bangladesh, STU, Sri Lankan Tamils in the UK, ITU, Indian Telugu in the UK, GIH, Gujarati Indians in Houston TX USA.
(TIF)

S4 Fig Distributions of the sizes of small indels in STR regions with an STR motif size of 1 bp. **(a)** Platinum NA12877, **(b)** Platinum NA12878, **(c)** GIAB HG002, **(d)** GIAB HG003, **(e)** GIAB HG004, **(f)** GIAB HG005, **(g)** GIAB HG006, and **(h)** GIAB HG007. Deletions and insertions are shown in blue and green, respectively.
(TIF)

S5 Fig Distributions of the sizes of small indels in STR regions with an STR motif size of 2 bp. **(a)** Platinum NA12877, **(b)** Platinum NA12878, **(c)** GIAB HG002, **(d)** GIAB HG003, **(e)** GIAB HG004, **(f)** GIAB HG005, **(g)** GIAB HG006, and **(h)** GIAB HG007. Deletions and insertions are shown in blue and green, respectively.
(TIF)

S6 Fig Distributions of the sizes of small indels in STR regions with STR motif size 3bp. **(a)** Platinum NA12877, **(b)** Platinum NA12878, **(c)** GIAB HG002, **(d)** GIAB HG003, **(e)** GIAB HG004, **(f)** GIAB HG005, **(g)** GIAB HG006, **(h)** GIAB HG007. Deletions and insertions are shown as blue and green, respectively.
(TIF)

S7 Fig Distributions of the sizes of small indels in STR regions with an STR motif of size 4 bp. **(a)** Platinum NA12877, **(b)** Platinum NA12878, **(c)** GIAB HG002, **(d)** GIAB HG003, **(e)** GIAB HG004, **(f)** GIAB HG005, **(g)** GIAB HG006, and **(h)** GIAB HG007. Deletions and insertions are shown in blue and green, respectively.
(TIF)

S8 Fig Distributions of the sizes of small indels in STR regions with an STR motif size of 5 bp. **(a)** Platinum NA12877, **(b)** Platinum NA12878, **(c)** GIAB HG002, **(d)** GIAB HG003, **(e)** GIAB HG004, **(f)** GIAB HG005, **(g)** GIAB HG006, and **(h)** GIAB HG007. Deletions and insertions are shown in blue and green, respectively.
(TIF)

S9 Fig Distributions of the sizes of small indels in STR regions with an STR motif size of 6 bp. **(a)** Platinum NA12877, **(b)** Platinum NA12878, **(c)** GIAB HG002, **(d)** GIAB HG003, **(e)** GIAB HG004, **(f)** GIAB HG005, **(g)** GIAB HG006, and **(h)** GIAB HG007. Deletions and insertions are shown in blue and green, respectively.
(TIF)

S10 Fig Distributions of copy numbers of motif-size 1 bp STRs containing variants. **(a)** Platinum NA12877, **(b)** Platinum NA12878, **(c)** GIAB HG002, **(d)** GIAB HG003, **(e)** GIAB HG004, **(f)** GIAB HG005, **(g)** GIAB HG006, and **(h)** GIAB HG007.
(TIF)

S11 Fig Distributions of copy numbers of motif-size 2 bp STRs containing variants. **(a)** Platinum NA12877, **(b)** Platinum NA12878, **(c)** GIAB HG002, **(d)** GIAB HG003, **(e)** GIAB HG004, **(f)** GIAB HG005, **(g)** GIAB HG006, and **(h)** GIAB HG007.
(TIF)

S12 Fig Distributions of copy numbers of motif-size 3 bp STRs containing variants. **(a)** Platinum NA12877, **(b)** Platinum NA12878, **(c)** GIAB HG002, **(d)** GIAB HG003, **(e)** GIAB HG004, **(f)** GIAB HG005, **(g)** GIAB HG006, and **(h)** GIAB HG007.
(TIF)

S13 Fig Distributions of copy numbers of motif-size 4 bp STRs containing variants. **(a)** Platinum NA12877, **(b)** Platinum NA12878, **(c)** GIAB HG002, **(d)** GIAB HG003, **(e)** GIAB HG004, **(f)** GIAB HG005, **(g)** GIAB HG006, and **(h)** GIAB HG007.
(TIF)

S14 Fig Distributions of copy numbers of motif-size 5 bp STRs containing variants. **(a)** Platinum NA12877, **(b)** Platinum NA12878, **(c)** GIAB HG002, **(d)** GIAB HG003, **(e)** GIAB HG004, **(f)** GIAB HG005, **(g)** GIAB HG006, and **(h)** GIAB HG007.
(TIF)

S15 Fig Distributions of copy numbers of motif-size 6 bp STRs containing variants. **(a)** Platinum NA12877, **(b)** Platinum NA12878, **(c)** GIAB HG002, **(d)** GIAB HG003, **(e)** GIAB HG004, **(f)** GIAB HG005, **(g)** GIAB HG006, and **(h)** GIAB HG007.
(TIF)

S1 Table The discordant breakpoint ambiguous, unnormalized indel annotations from VarSCAT and UPS-indel of Platinum Genomes sample NA12878. (XLSX)

S2 Table The discordant breakpoint ambiguous, complex indel annotations from VarSCAT and UPS-indel of Platinum Genomes sample NA12878. (XLSX)

S3 Table The running time and maximum memory usage of VarSCAT and UPS-indel from samples of Genome in a Bottle HG002-HG007 and Platinum Genomes NA12877 and NA12878. The running times were measured with Intel Xeon Gold 6230 CPU @ 2.10GHz.
(XLSX)

S1 File Supplementary methods descriptions. (DOCX)
