# Supplementary figures and images for: VarSCAT: A computational tool for sequence context annotations of genomic variants
Source: PLoS Comput Biol. 2023 Aug 11;19(8):e1010727. doi: 10.1371/journal.pcbi.1010727 (PMC10446208; doi:10.1371/journal.pcbi.1010727)

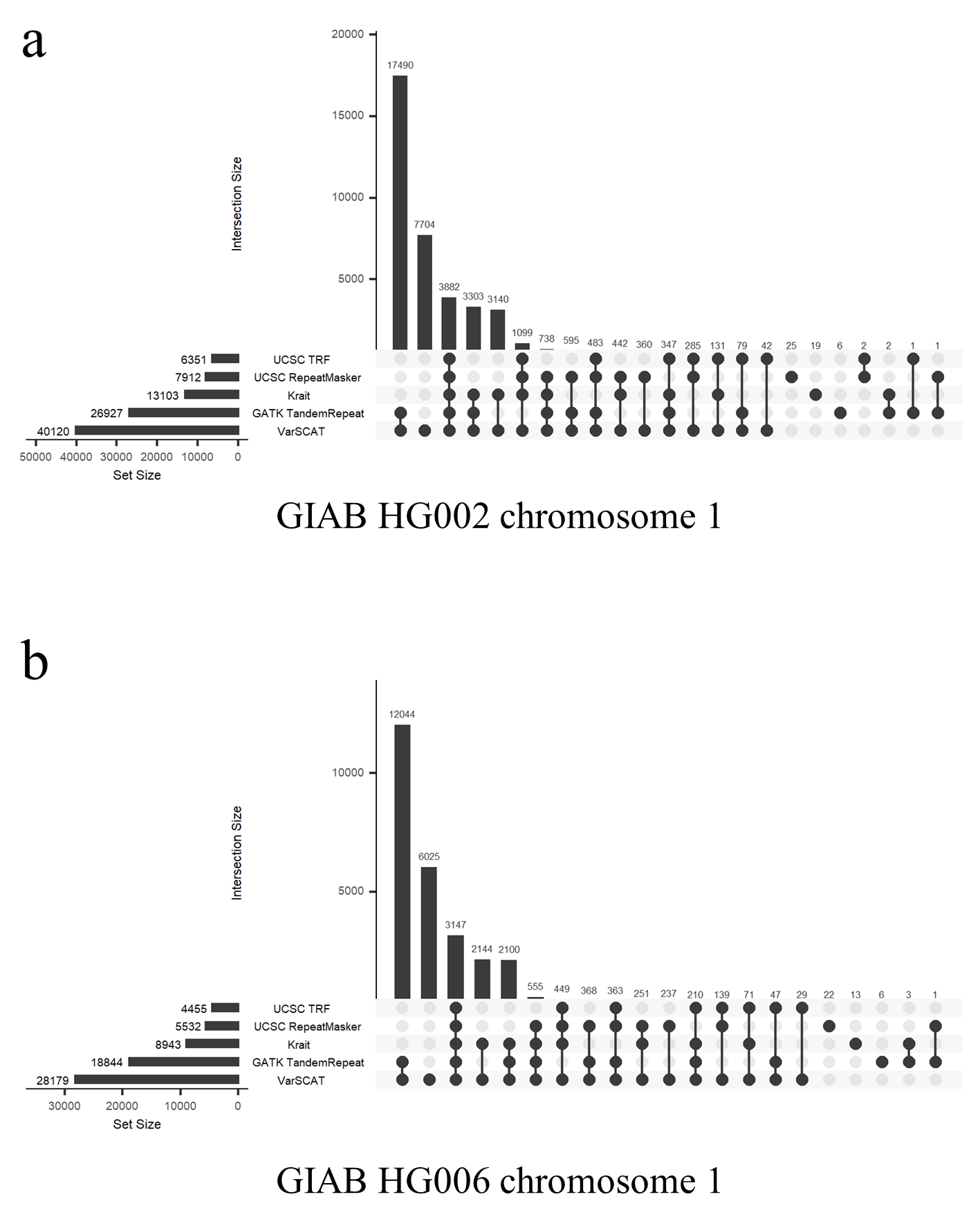

Supplement: S1 Fig — The benchmarking was performed with small variants of chromosome 1 of (a) GIAB HG002, and (b) GIAB HG006. The numbers are the counts of variants annotated by each tool. (TIF) [file pcbi.1010727.s001.TIF]

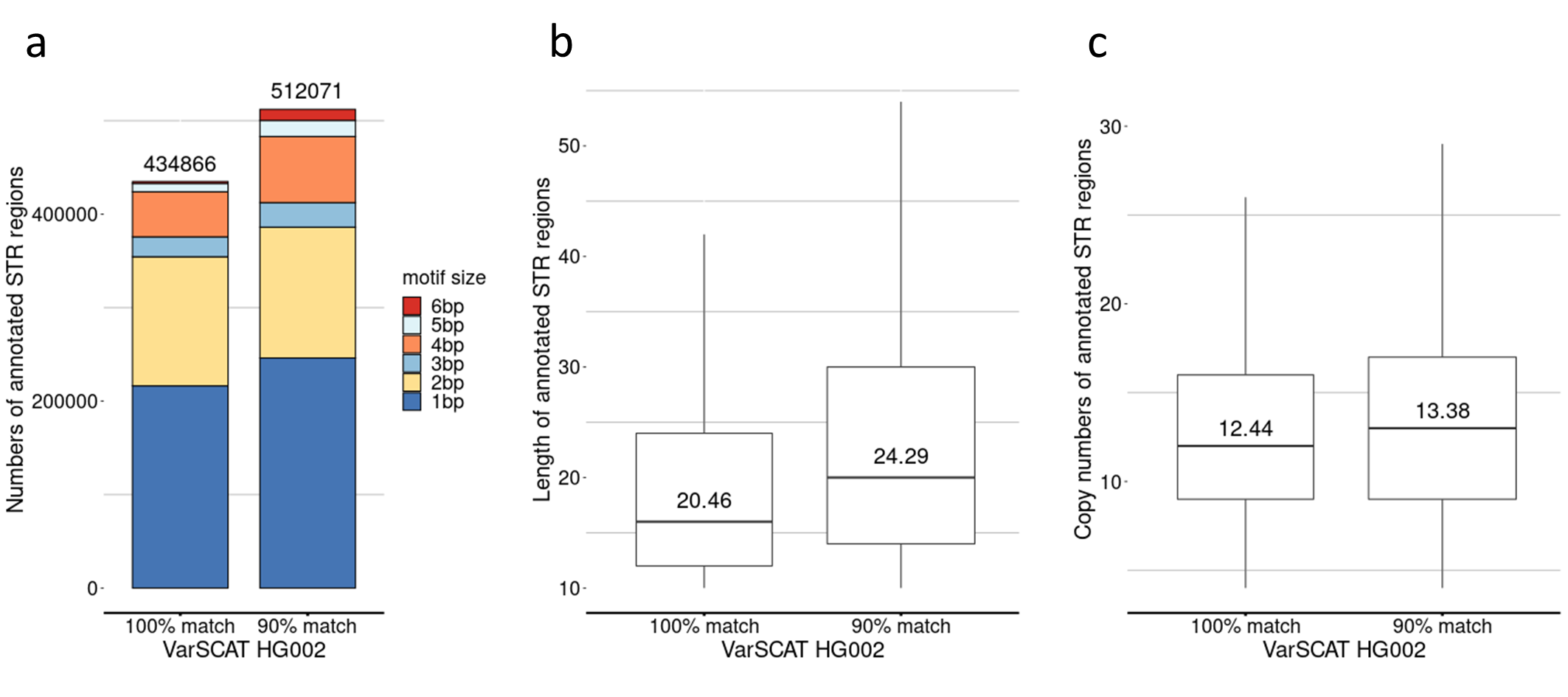

Supplement: S2 Fig — (a) the barplots of numbers of annotated STR regions. Different colors indicated the different sizes of repeat motifs and the numbers on top of each bar were the total numbers of annotated STR regions, (b) the boxplots of length of annotated STR regions. The numbers in the middle of each box were the average lengths of annotated STR regions, and (c) the boxplots of copy number of annotated STR regions. The numbers in the middle of each box were the average copy numbers of annotated STR regions. (TIF) [file pcbi.1010727.s002.tif]

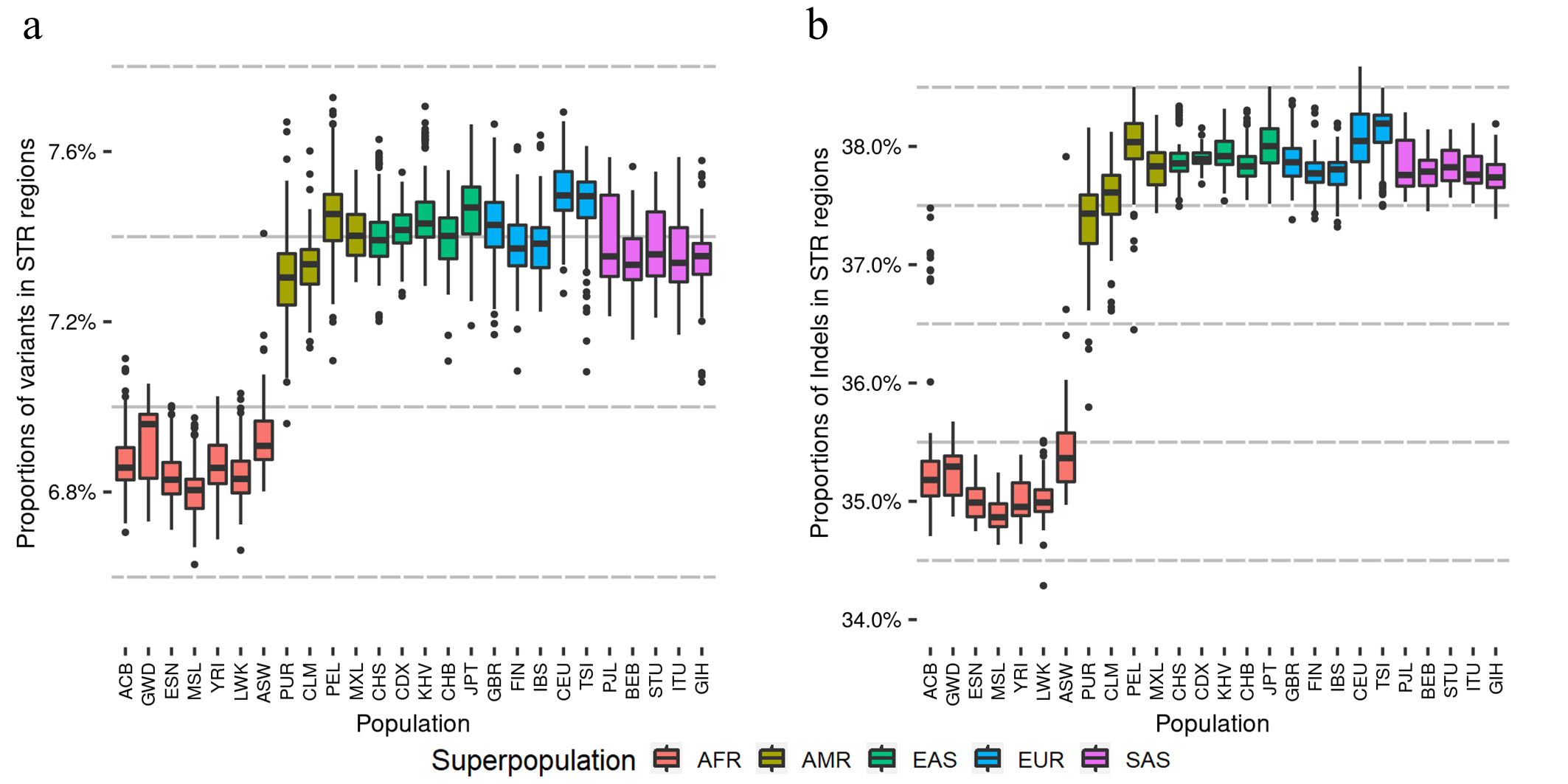

Supplement: S3 Fig — (a) The proportions of small variants in STR regions and (b) the proportions of small indels in STR regions. ACB, African Caribbean in Barbados, GWD, Gambian in Western Division Mandinka, ESN, Esan in Nigeria, MSL, Mende in Sierra Leone, YRI, Yoruba in Ibadan Nigeria, LWK, Luhya in Webuye Kenya, ASW, People with African Ancestry in Southwest USA, PUR, Puerto Ricans in Puerto Rico, CLM, Colombians in Medellin Colombia, PEL, Peruvians in Lima Peru, MXL, People with Mexican Ancestry in Los Angeles CA USA, CHS, Southern Han Chinese, CDX, Chinese Dai in Xishuangbanna China, KHV, Kinh in Ho Chi Minh City Vietnam, CHB, Han Chinese in Beijing, China, JPT, Japanese in Tokyo Japan, GBR, British in England and Scotland, FIN, Finnish in Finland, IBS, Iberian Populations in Spain, CEU, Utah residents with Northern and Western European ancestry, TSI, Tuscans in Italy, PJL, Punjabis in Lahore Pakistan, BEB, Bengalis in Bangladesh, STU, Sri Lankan Tamils in the UK, ITU, Indian Telugu in the UK, GIH, Gujarati Indians in Houston TX USA. (TIF) [file pcbi.1010727.s003.tif]

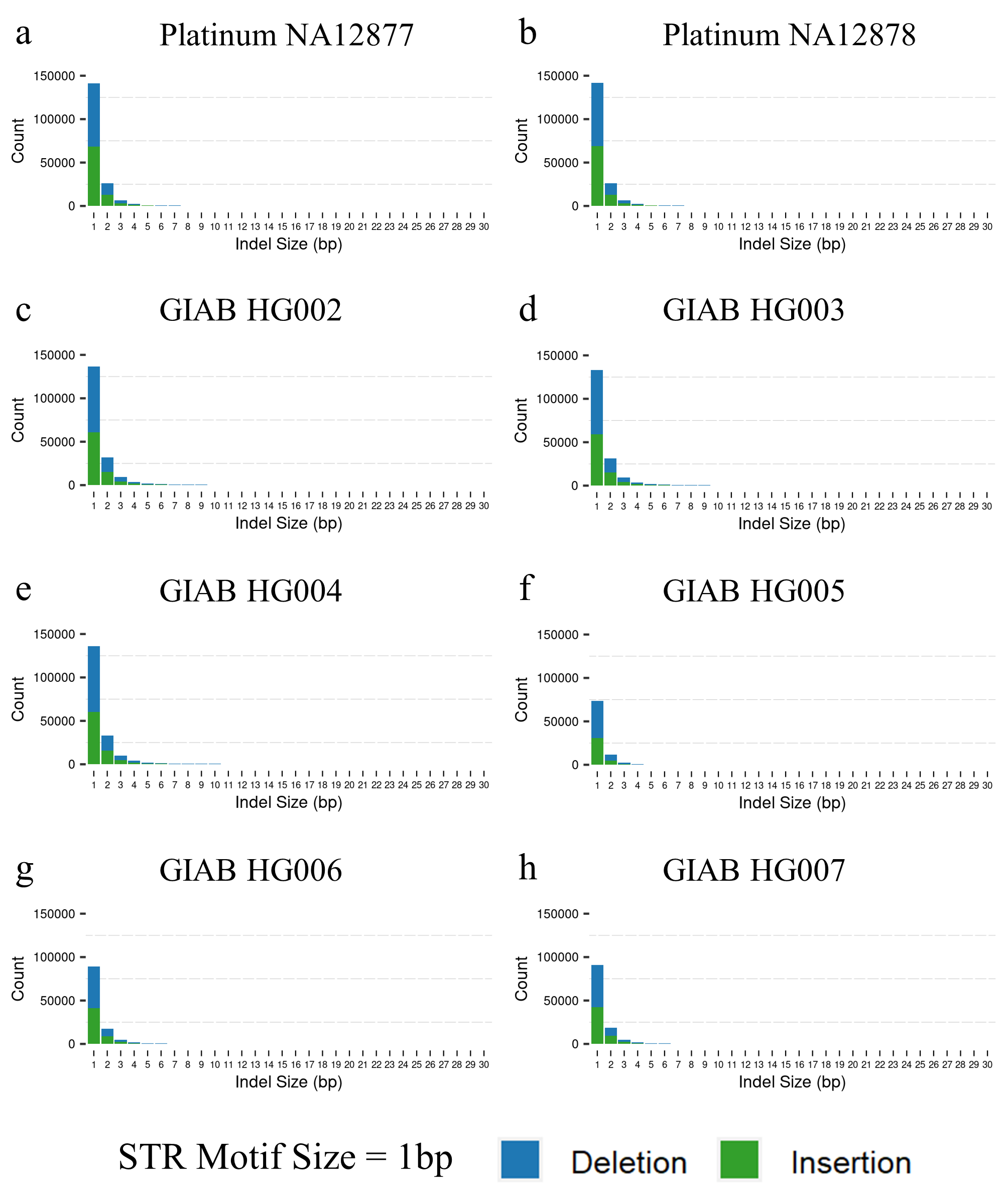

Supplement: S4 Fig — (a) Platinum NA12877, (b) Platinum NA12878, (c) GIAB HG002, (d) GIAB HG003, (e) GIAB HG004, (f) GIAB HG005, (g) GIAB HG006, and (h) GIAB HG007. Deletions and insertions are shown in blue and green, respectively. (TIF) [file pcbi.1010727.s004.TIF]

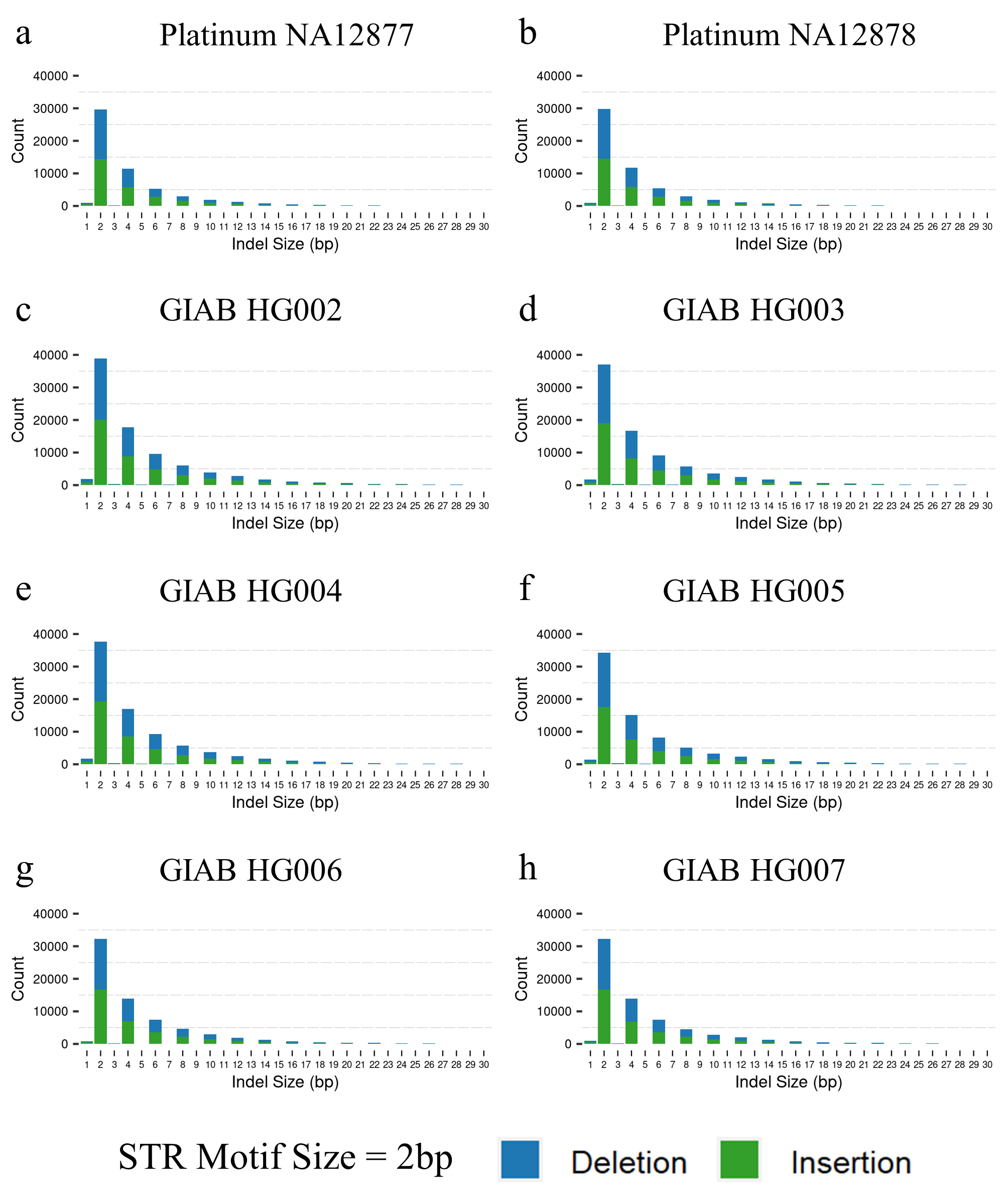

Supplement: S5 Fig — (a) Platinum NA12877, (b) Platinum NA12878, (c) GIAB HG002, (d) GIAB HG003, (e) GIAB HG004, (f) GIAB HG005, (g) GIAB HG006, and (h) GIAB HG007. Deletions and insertions are shown in blue and green, respectively. (TIF) [file pcbi.1010727.s005.TIF]

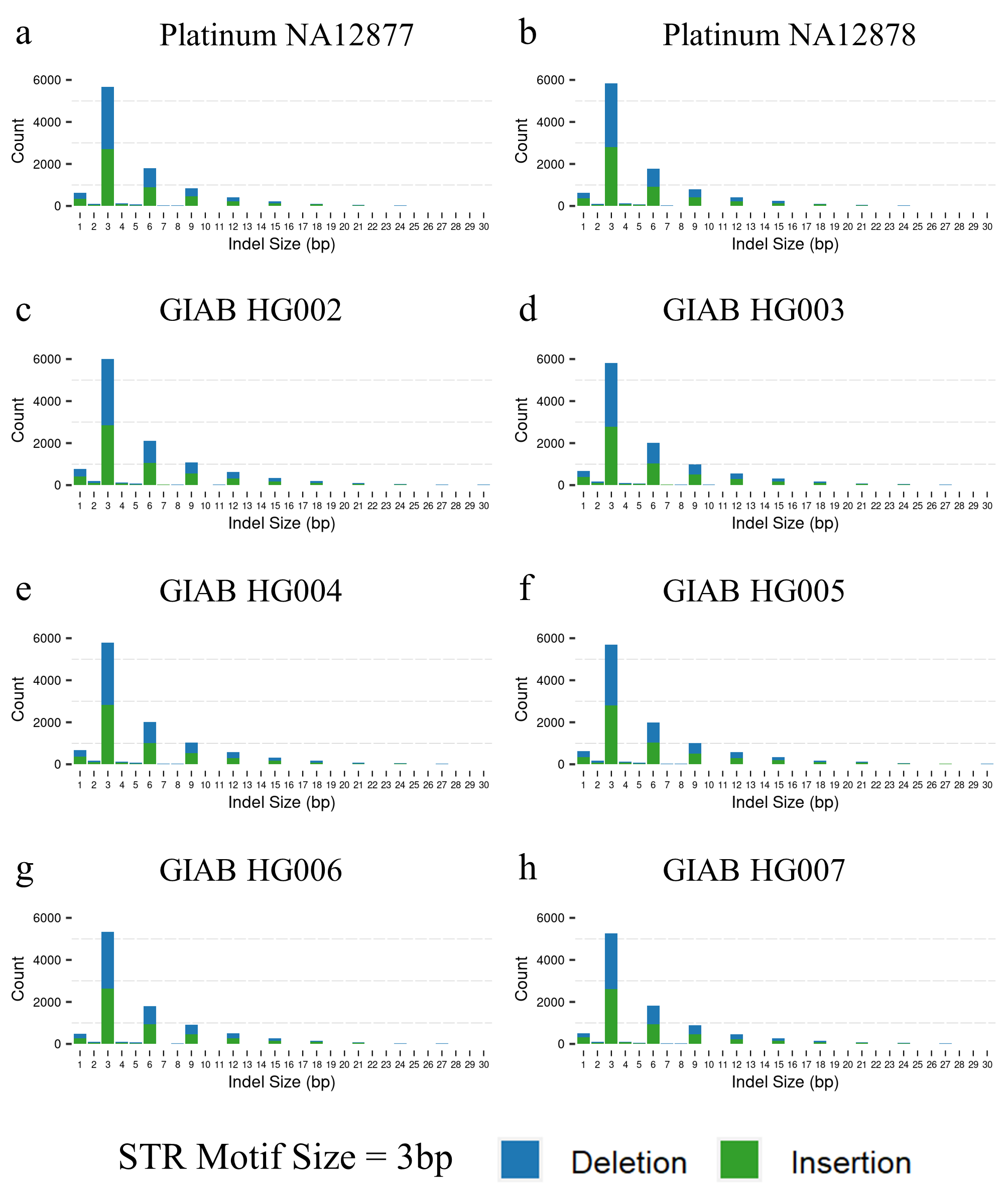

Supplement: S6 Fig — (a) Platinum NA12877, (b) Platinum NA12878, (c) GIAB HG002, (d) GIAB HG003, (e) GIAB HG004, (f) GIAB HG005, (g) GIAB HG006, (h) GIAB HG007. Deletions and insertions are shown as blue and green, respectively. (TIF) [file pcbi.1010727.s006.TIF]

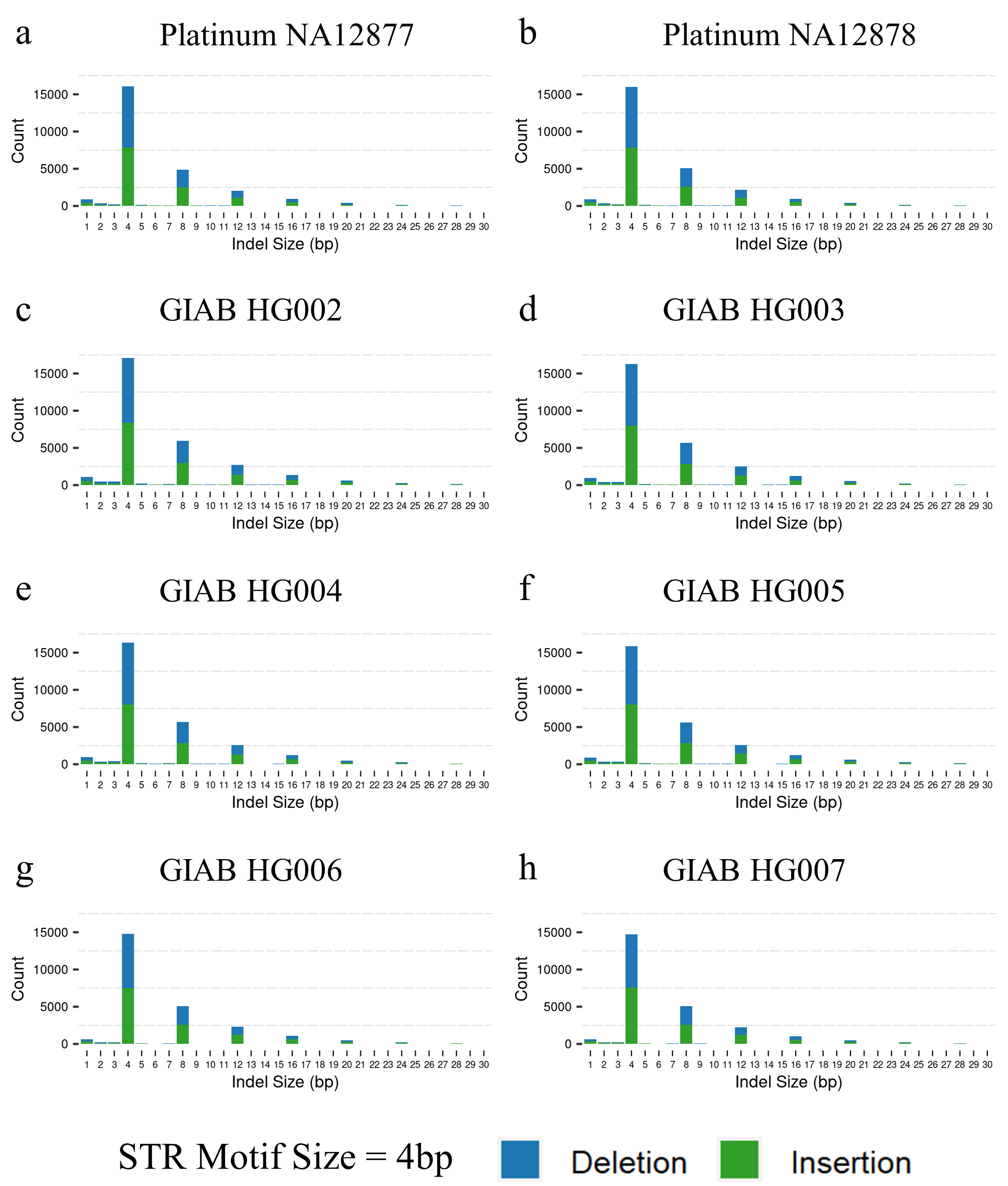

Supplement: S7 Fig — (a) Platinum NA12877, (b) Platinum NA12878, (c) GIAB HG002, (d) GIAB HG003, (e) GIAB HG004, (f) GIAB HG005, (g) GIAB HG006, and (h) GIAB HG007. Deletions and insertions are shown in blue and green, respectively. (TIF) [file pcbi.1010727.s007.TIF]

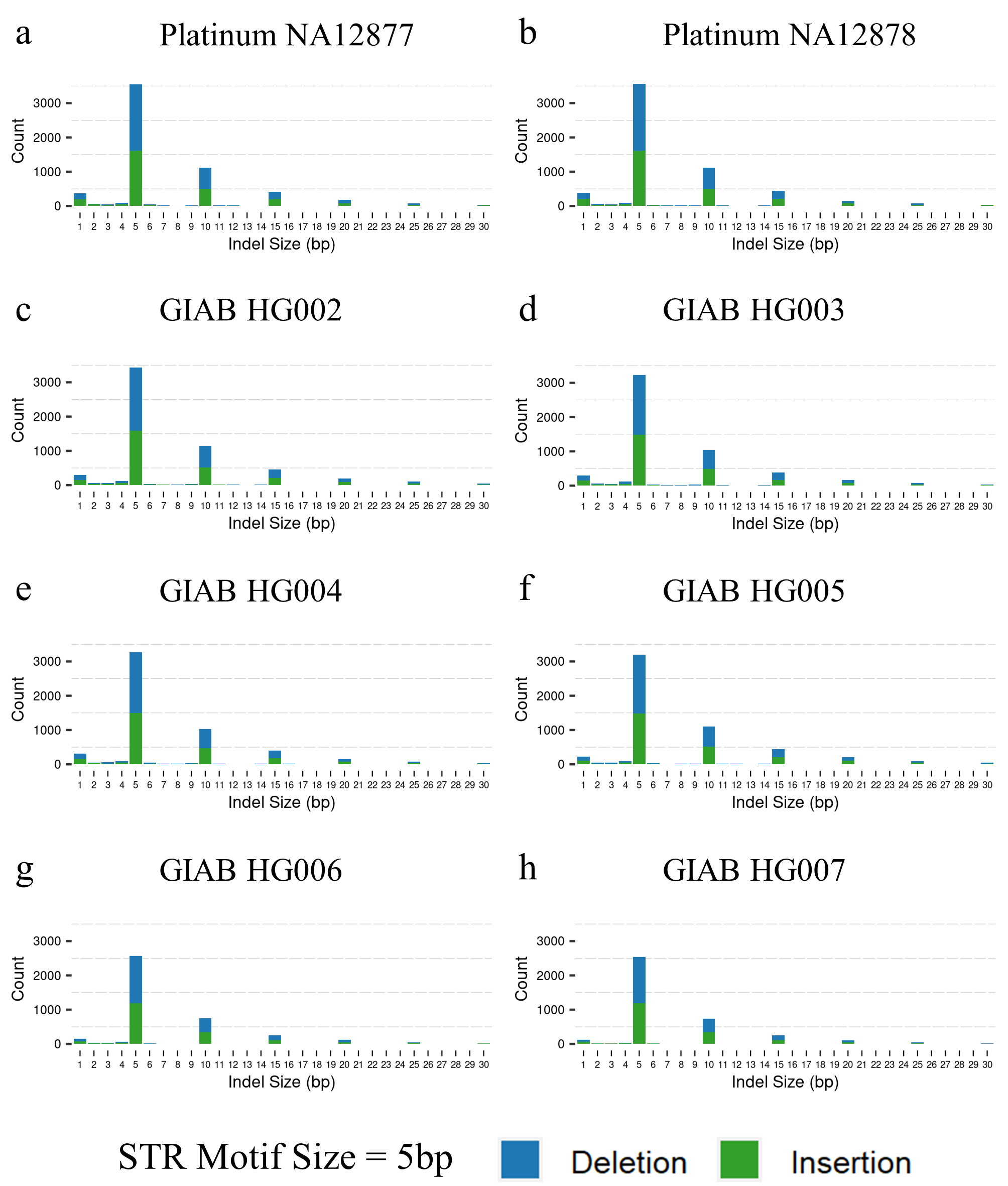

Supplement: S8 Fig — (a) Platinum NA12877, (b) Platinum NA12878, (c) GIAB HG002, (d) GIAB HG003, (e) GIAB HG004, (f) GIAB HG005, (g) GIAB HG006, and (h) GIAB HG007. Deletions and insertions are shown in blue and green, respectively. (TIF) [file pcbi.1010727.s008.TIF]

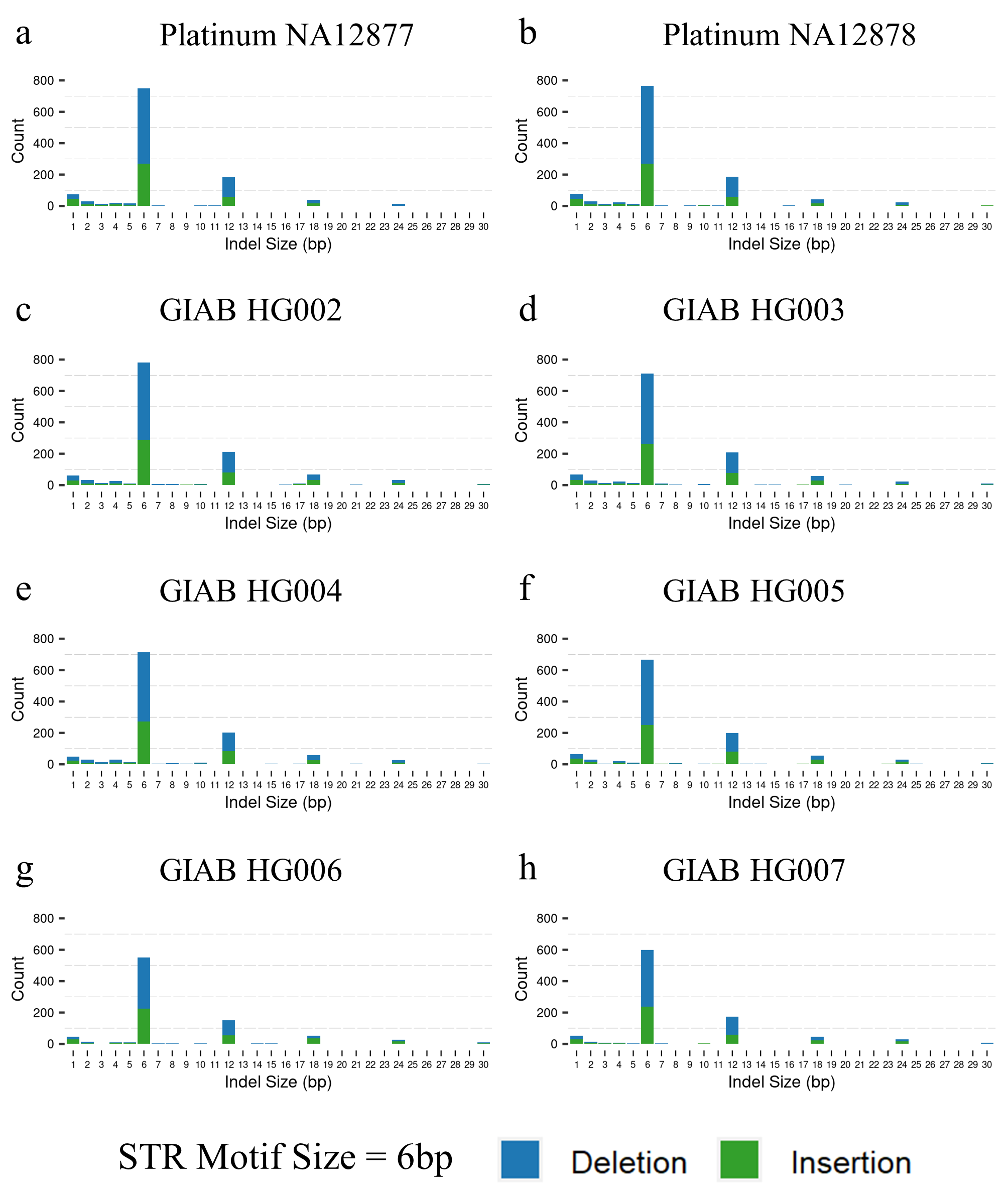

Supplement: S9 Fig — (a) Platinum NA12877, (b) Platinum NA12878, (c) GIAB HG002, (d) GIAB HG003, (e) GIAB HG004, (f) GIAB HG005, (g) GIAB HG006, and (h) GIAB HG007. Deletions and insertions are shown in blue and green, respectively. (TIF) [file pcbi.1010727.s009.TIF]

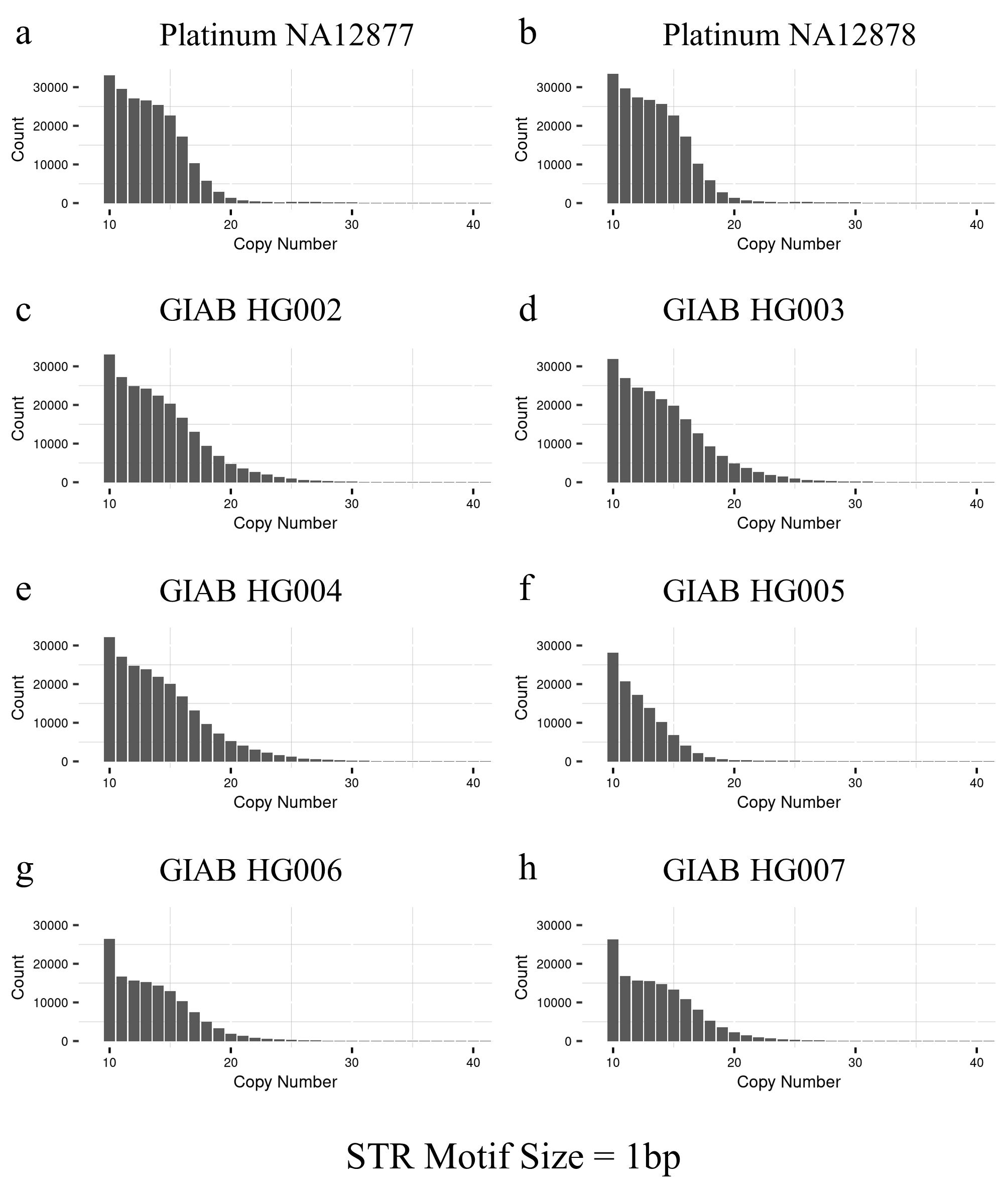

Supplement: S10 Fig — (a) Platinum NA12877, (b) Platinum NA12878, (c) GIAB HG002, (d) GIAB HG003, (e) GIAB HG004, (f) GIAB HG005, (g) GIAB HG006, and (h) GIAB HG007. (TIF) [file pcbi.1010727.s010.TIF]

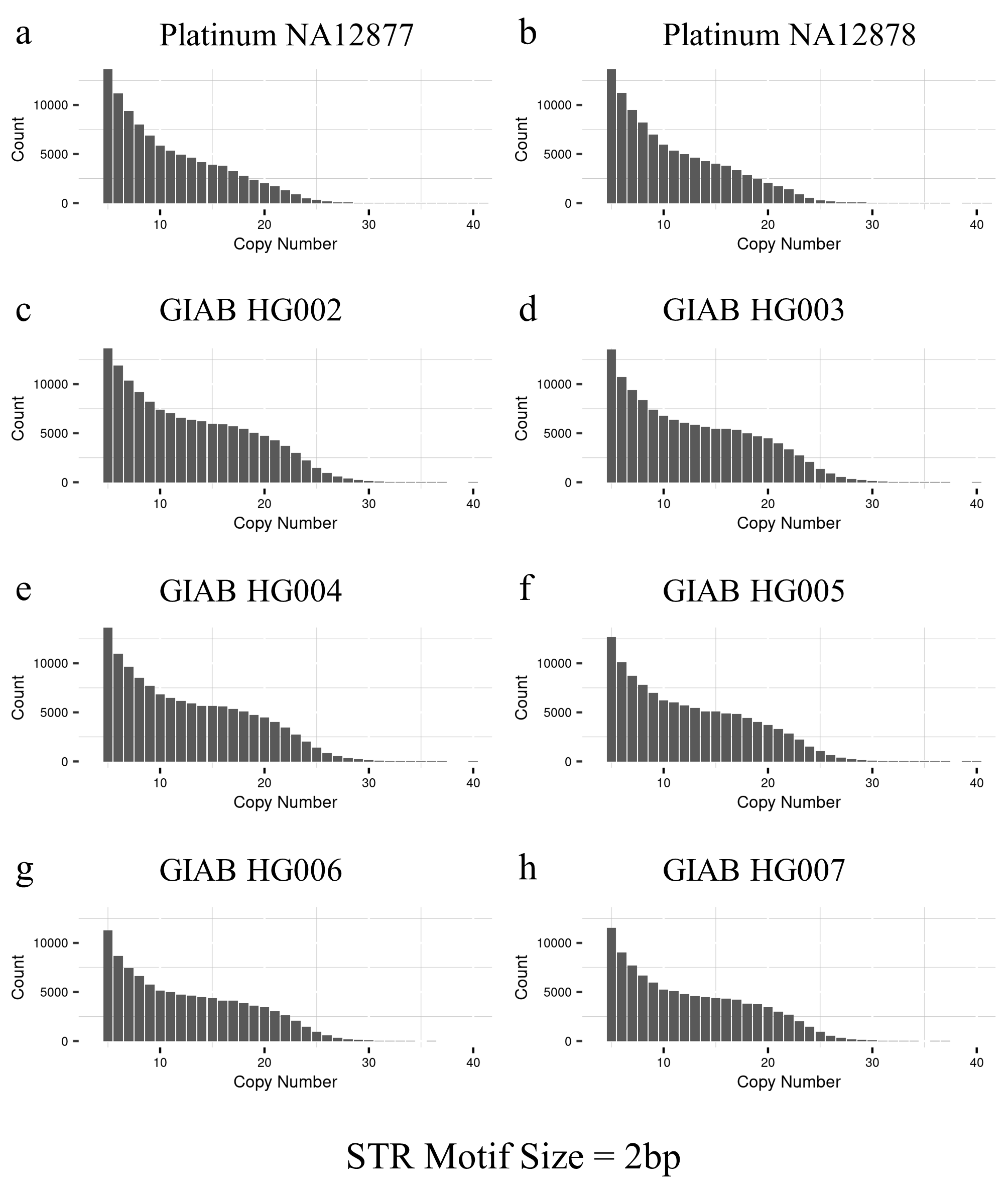

Supplement: S11 Fig — (a) Platinum NA12877, (b) Platinum NA12878, (c) GIAB HG002, (d) GIAB HG003, (e) GIAB HG004, (f) GIAB HG005, (g) GIAB HG006, and (h) GIAB HG007. (TIF) [file pcbi.1010727.s011.TIF]

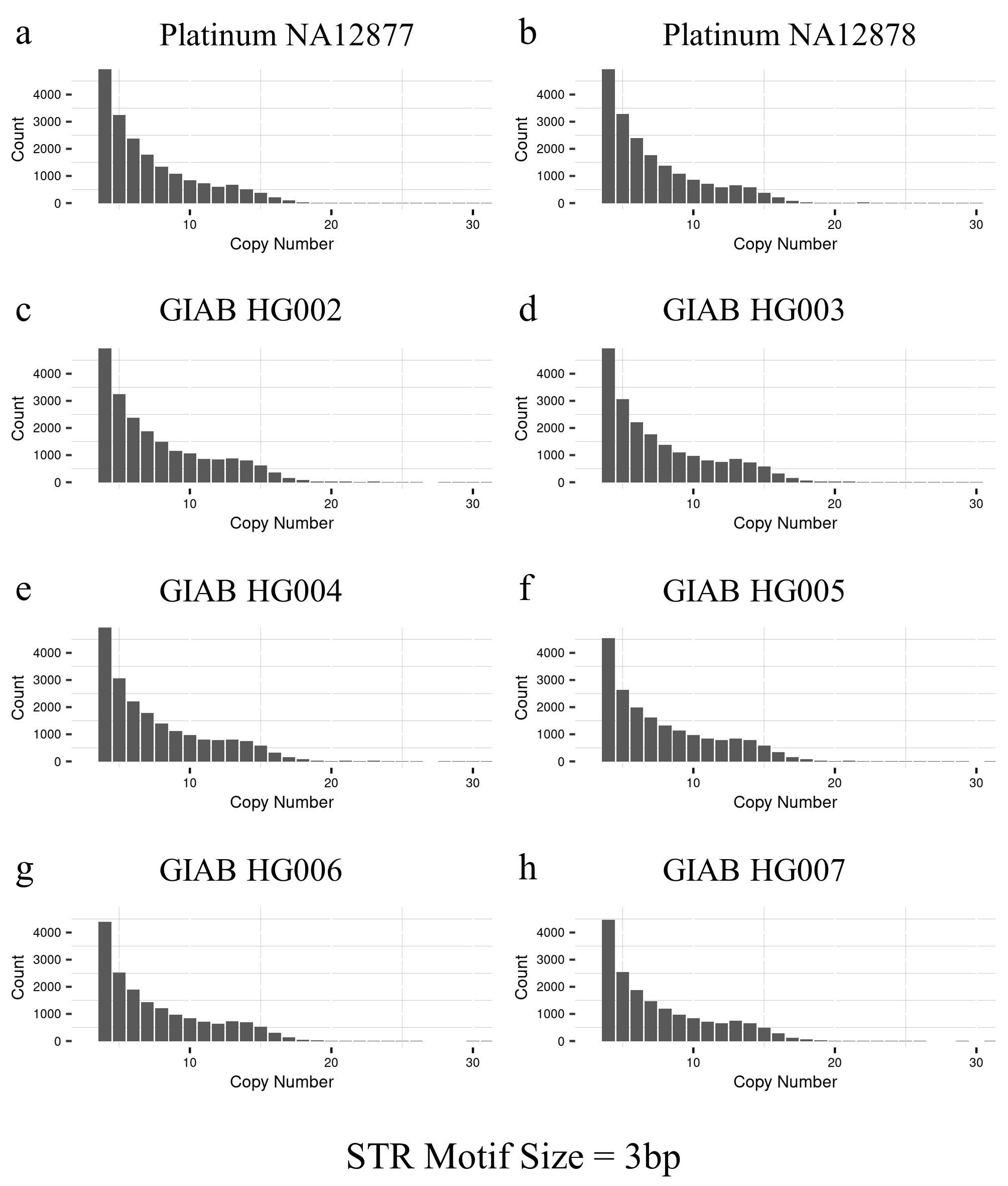

Supplement: S12 Fig — (a) Platinum NA12877, (b) Platinum NA12878, (c) GIAB HG002, (d) GIAB HG003, (e) GIAB HG004, (f) GIAB HG005, (g) GIAB HG006, and (h) GIAB HG007. (TIF) [file pcbi.1010727.s012.TIF]

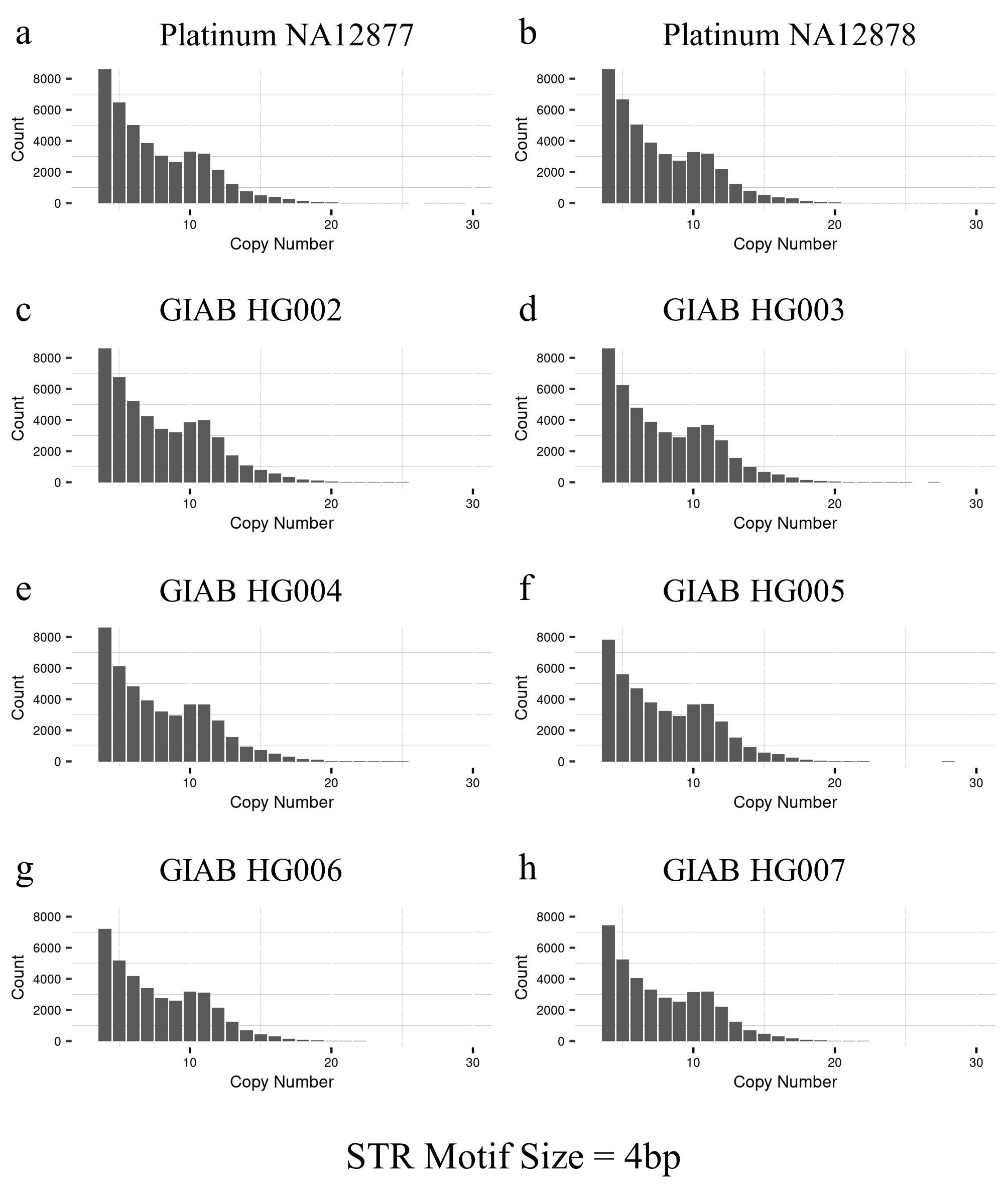

Supplement: S13 Fig — (a) Platinum NA12877, (b) Platinum NA12878, (c) GIAB HG002, (d) GIAB HG003, (e) GIAB HG004, (f) GIAB HG005, (g) GIAB HG006, and (h) GIAB HG007. (TIF) [file pcbi.1010727.s013.TIF]

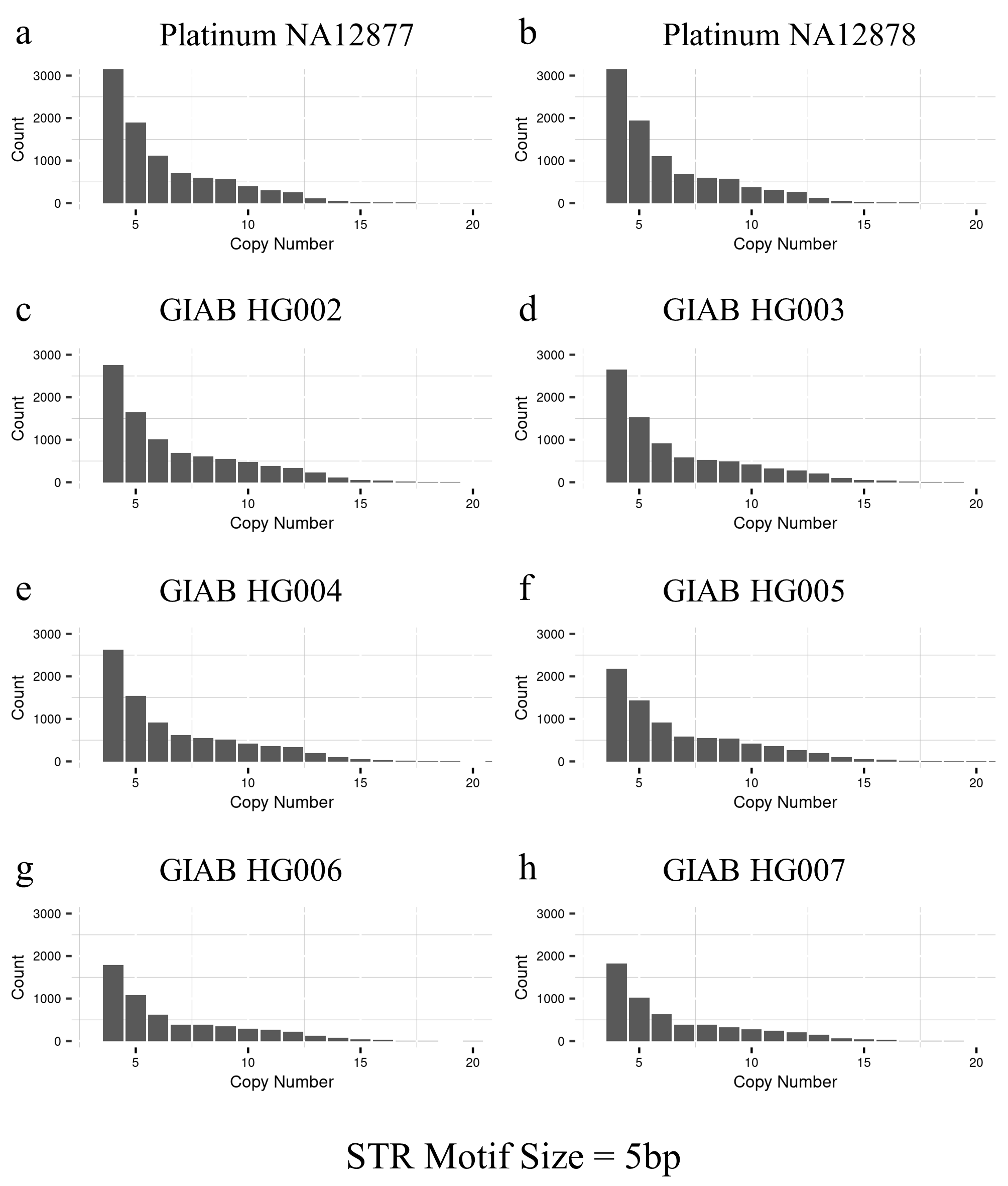

Supplement: S14 Fig — (a) Platinum NA12877, (b) Platinum NA12878, (c) GIAB HG002, (d) GIAB HG003, (e) GIAB HG004, (f) GIAB HG005, (g) GIAB HG006, and (h) GIAB HG007. (TIF) [file pcbi.1010727.s014.TIF]

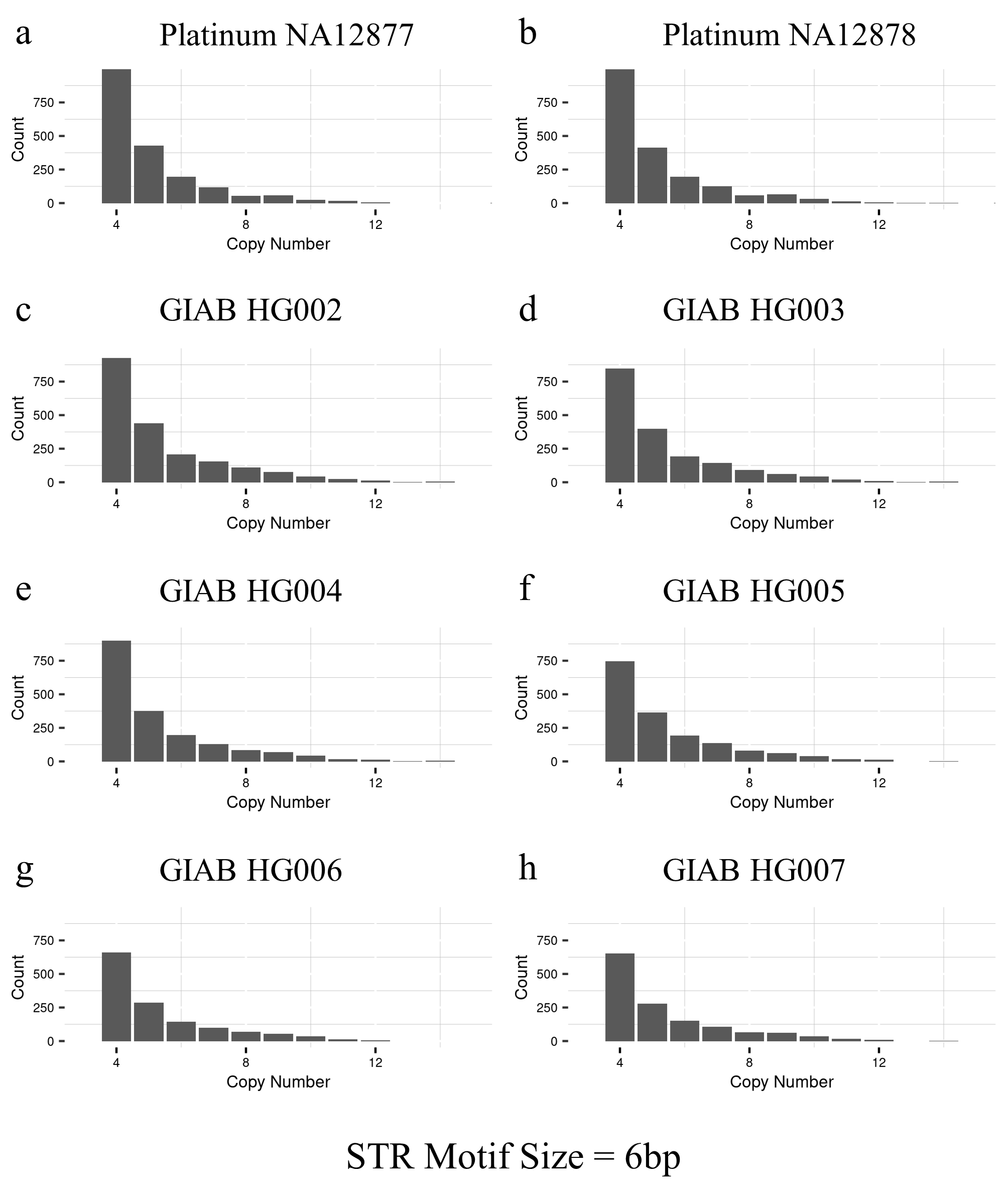

Supplement: S15 Fig — (a) Platinum NA12877, (b) Platinum NA12878, (c) GIAB HG002, (d) GIAB HG003, (e) GIAB HG004, (f) GIAB HG005, (g) GIAB HG006, and (h) GIAB HG007. (TIF) [file pcbi.1010727.s015.TIF]
