# Supplementary material for: VarSCAT: A computational tool for sequence context annotations of genomic variants
Source: PLoS Comput Biol. 2023 Aug 11;19(8):e1010727. doi: 10.1371/journal.pcbi.1010727 (PMC10446208; doi:10.1371/journal.pcbi.1010727)
Supplement: S1 File — (DOCX) [file pcbi.1010727.s019.docx]

**VarSCAT: A computational tool for sequence context annotations of genomic variants**

Ning Wang^*^, Sofia Khan, Laura L. Elo^*^

*To whom correspondence should be addressed

**Section S1: Parameter settings of VarSCAT in this study**

For the adjacent sequence annotation module, we used the 5’-aligned positions, 3’-aligned positions, and 3’ edge positions of variants to analyze breakpoint ambiguities and duplicate of variants by turning on ‘--LRP’ parameters. For the tandem repeat annotation module, due to the various subjective definitions of short tandem repeats (STRs) used in different studies, especially for imperfect STRs, the annotation results could vary widely. Thus, in our study, we used only perfect STRs with motif sizes of 1 – 6 bp and set the minimum length of a STR region to 10 bp. We also set minimum copy number to ten for mononucleotide STRs, five for dinucleotide STRs, and four for tri- to hexanucleotide STRs. We annotated the candidate perfect STRs by setting the parameters of VarSCAT at ‘--min_unit 1’, ‘--max_unit 6’, ‘--min_time 4’, ‘--match 1’, ‘--mismatch -1’, ‘--gap -2’, ‘--similarity 100’, ‘--gap_tolerate 0’, ‘--min_score 10’. Other VarSCAT parameters were set to defaults. The only exceptions of imperfect STR region annotations were the benchmarking of VarSCAT for imperfect STR regions and comparison of VarSCAT perfect and imperfect STR regions annotations (Results of S1 and S2 Figs). For imperfect STR regions, the motif sizes and corresponding copy numbers were the same as perfect STR region annotations. However, it allowed at least 75% similarity between each repeat unit and the maximum gap between repeat units was one repeat motif size. The total match percentage of an imperfect STR region should be 90% to its repeat motif. We annotated the candidate imperfect STRs by setting the parameters of VarSCAT at ‘--min_unit 1’, ‘--max_unit 6’, ‘--min_time 4’, ‘--match 1’, ‘--mismatch -1’, ‘--gap -2’, ‘--similarity 75’, ‘--gap_tolerate -1’, ‘--min_score 10’, and ‘--min_match_per 90’. Other VarSCAT parameters were set to defaults. The imperfect STR region should have at least 10 alignment sum score according to Equation. 1 in main text “Methods and Materials” section, with match score 1, mismatch score -1, and gap penalty -2. VarSCAT was run with Python version 3.6.8. In our analysis, we treated a variant as a deletion if the number of nucleotides in the ‘REF’ column of the variant call format (VCF) file was larger than the number of nucleotides in the ‘ALT’ column of the VCF file, as an insertion if the number of nucleotides in the ‘REF’ column of the VCF file was smaller than the number of nucleotides in the ‘ALT’ column of the VCF file, and as a single nucleotide variant (SNV) or multi-nucleotide variant (MNV) if the number of nucleotides in the ‘REF’ column of the VCF file was equal to the number of nucleotides in ‘ALT’ column of the VCF file.

**Section S2: Data processing for benchmarking of VarSCAT**

For STR region variant annotations, benchmarking was performed with variants of chromosome 1 of Genome of a Bottle (GIAB) sample HG002 and HG006 v4.2.1 (the son of an Ashkenazi Jewish ancestry trio and the father of a Han Chinese ancestry trio). The VCF files were downloaded from the GIAB FTP site (https://ftp-trace.ncbi.nlm.nih.gov/giab/ftp/release/) and split into per-chromosome VCF files with SnpSift (version 5.0d). The reference sequence of GRCh38 was obtained from the GIAB FTP site to ensure that the version of the reference sequence file of chromosome 1 was consistent with the VCF files (https://ftp-trace.ncbi.nlm.nih.gov/giab/ftp/release/references/GRCh38/). The reference sequence file was indexed using Samtools (v1.11), BWA (0.7.17-r1198-dirty), and GATK (v4.1.9.0). The VCF files for chromosome 1 of GIAB HG002 and HG006 were first processed using the GATK ‘LeftAlignAndTrimVariants’ module with the ‘--split-multi-allelics’ function to split potential multiallelic variants into biallelic variants. The vt (v0.57712) [1] normalization function was then applied to the VCF files to left align all variants.

For the methods that directly annotate variants in STR regions with a reference genome, VarSCAT was applied the parameters and settings mentioned in Section S1. GATK ‘TandemRepeat’ function to annotate the biallelic and normalized VCF files of GIAB HG002 and HG006. We selected the GATK ‘TandemRepeat’ STR annotations were selected based on the ‘STR’ flag, extracted the motifs from the ‘RU’ flag, and extracted the STR copy numbers on the reference sequence from the first integer of the ‘RPA’ flag. We selected the variants annotated by GATK ‘TandemRepeat’ as STR variants with a custom R script that met our criteria, as mentioned in Section S1. Due to GATK ‘TandemRepeat’ can only annotate perfect STR regions, so the same annotations made by GATK ‘TandemRepeat’ was applied to benchmarking of both perfect and imperfect STRs.

For the method that detect STR regions with a reference genome and then use the annotation tool to annotate variants, we used the Krait (v.1.3.3, https://github.com/lmdu/krait) ‘SSRs’ function to search for perfect STRs with parameters of a minimum of ten ‘repeats’ for mononucleotide STRs, five ‘repeats’ for dinucleotide STRs, and four ‘repeats’ for tri- to hexanucleotide STRs to annotate chromosome 1 of the human reference genome GRCh38 with an FASTA file (https://hgdownload.soe.ucsc.edu/goldenPath/hg38/chromosomes/). For imperfect STRs, we combined Krait perfect STR annotations from ‘SSRs’ function and imperfect STR annotations from ‘iSSRs’ function. The parameters for ‘iSSRs’ function was default which was ‘Min seed repeats’ 3, ‘Min seed length’ 8, ‘Max continuous edits’ 3, ‘Mismatch penalty’ 1, ‘Gap penalty’ 2, and ‘Min require score’ 10. The minimum copy numbers for each size of repeat motif were the same as perfect STRs and the imperfect STRs were further filtered to keep STRs with at least 90% matches (calculated by ‘match’/‘length’, which were two annotations of Krait for each STR). For the methods that download ready-made STR annotations and then use the annotation tool to annotate variants, we used TRF and RepeatMasker which were represented by the ‘Simple Repeat’ and ‘RepeatMasker’ tracks of chromosome 1 of human reference hg38 from the UCSC genome browser (download date: 11 February 2022), respectively. We selected TRF and RepeatMasker records (only records flagged by ‘Simple_repeat’ were selected) with STR motif sizes of 1–6 bp and a minimum of ten copy numbers for mononucleotide STRs, five copy numbers for dinucleotide STRs, and four copy numbers for tri- to hexanucleotide STRs. For TRF, the copy numbers were determined from the ‘CopyNum’ column of the track file, and for RepeatMasker, the copy numbers were calculated using the STR regions (‘repEnd’ and ‘repStart’ columns of the track files) divided by the sizes of STR motifs (‘repName’ column of the track file). For the benchmarking with perfect TRF and RepeatMasker STRs, we limited the records to STRs without any gaps or mismatches. For TRF, the perfect STRs were selected based on the ‘perMatch’ column of each STR with a value of 100 and the imperfect STRs were selected with a value of at least 90. For RepeatMasker, the perfect STRs were selected based on the sum scores of columns ‘milliDiv’, ‘milliDel’, and ‘milliIns’ for each STR that was equal to zero and the imperfect STRs were selected with a value of at most 100 (according to the description on UCSC Genome Browser, these values are in ‘per thousand’ scale). The Krait, TRF and RepeatMasker results were then converted into a BED format file with three columns (chromosome, STR start position, and STR end position) using a custom R script. The BED files were then used as STR annotation resources for annotating the biallelic and normalized VCF files of GIAB HG002 and HG006 with ANNOVAR (version on ANNOVAR interface: $Date: 2019-10-24 00:05:27 -0400 (Thu, 24 Oct 2019)). For the annotation results from different methods, we used a custom R script to extract variants which were annotated STRs and assigned variant identifiers (IDs) which comprised the chromosome, position, REF string, and ALT string for each selected variant in the STR region. These IDs of variants were then used by UpSet plot (R [version 3.6.3] [2] package “UpSetR” version 1.4.0) to view the overlaps among the different annotation sets.

For variants breakpoint ambiguity annotation, UPS-indel (no version info available, download date: 08/03/2023, https://github.com/mshabbirhasan/ups-indel) was used to compare with VarSCAT. Insertions and deletions (indels) were selected using VCFtools (v0.1.17, --keep-only-indels, --recode) from eight high-confidence, human variant sets GIAB HG002-HG007, and Platinum Genomes NA12877 and NA12878. The concordance of indel breakpoint ambiguity annotations between VarSCAT and UPS-indel was performed by comparing 5’- and 3’-aligned positions of indels annotated by the two tools. The definition of 5’- and 3’-aligned positions for insertions were slightly differently between VarSCAT and UPS-indel. VarSCAT defined 5’- and 3’-aligned positions of an insertion were nucleotide coordinates that left to the inserted sequence, while UPS-indel’s definitions were nucleotide coordinates that right to the inserted sequence. The 5’- and 3’-aligned positions of insertions annotated by UPS-indel were modified accordingly. Due to a design limitation of UPS-indel that it cannot recognize chromosome coordinates start with “chr”, chromosome coordinates in all the VCF files and reference sequence files were modified using awk (awk '{gsub(/\chr/, "")}1', which can convert, for example. “chr1” to “1”). Venn Diagrams ((R [version 3.6.3], package “ggVennDiagram” version 1.2.0) were applied to show the concordance between annotations. To further verify the different annotations between VarSCAT and UPS-indel, we selected the discordant annotations between the two tools of Platinum Genomes NA12878, manually checked them, and listed them in S1 and S2 tables. The running time and maximum memory usage were measured with high performance computer clusters with Intel Xeon Gold 6230 CPU @ 2.10GHz. The workload manager Slurm [3] was used to record the running times and maximum memory usage (--seff jobID)

**Section S3: Data source, pre-processing, and analysis with VarSCAT**

We downloaded the VCF file (date: 9 January 2022) from the ClinVar database on the ClinVar FTP site (https://ftp.ncbi.nlm.nih.gov/pub/clinvar/vcf_GRCh38/). For the ClinVar variants, we first filtered out variants located in genome scaffolds and only kept variants of autosomes, sex chromosomes, and mitochondrial DNA. Thereafter, that we ran the remaining ClinVar variants through the VarSCAT adjacent sequence annotation module. The variants that contained nonvalid alternative alleles were filtered out from the VarSCAT results due to unknown patterns of variants.

We used the autosomes and chromosome X of Platinum Genome NA12877 and NA12878 in our analysis (https://github.com/Illumina/PlatinumGenomes/blob/master/files/2017-1.0.files, files: /hg38/small_variants/NA12877/NA12877.vcf.gz, /hg38/small_variants/NA12878/NA12878.vcf.gz). Due to a lack of variants for sex chromosomes, we included autosomes of GIAB HG002–HG0007 from v4.2.1 (trios of Ashkenazi Jewish and Han Chinese ancestry) in our analysis (https://ftp-trace.ncbi.nlm.nih.gov/giab/ftp/release/). The VCF files for high-confidence variant sets of Platinum Genome and GIAB samples were split into per-chromosome VCF files using SnpSift (version 5.0d), run through the VarSCAT adjacent sequence annotation module and annotated for perfect STRs by tandem repeat annotation module simultaneously using the parameters mentioned in Section S1.

For the 1000 Genomes Project data, we downloaded the sample genotypes integrated per-chromosome VCF files with the corresponding index files from the 1000 Genomes Project (http://ftp.1000genomes.ebi.ac.uk/vol1/ftp/data_collections/1000_genomes_project/release/20190312_biallelic_SNV_and_INDEL/). To reduce the memory consumption of VarSCAT, we applied BCFtools’ (version 1.5) ‘view’ and ‘query’ functions to the integrated VCF files and extracted the genotype information for each individual sample as a separate file, as well as produced VCF files without genotype information as the inputs for VarSCAT. We also created a custom Python script using the PyVCF package to extract the allele frequencies of the variants.

**Section S4: Data processing for the analysis of breakpoint-ambiguous indels and duplicates**

We first extracted the VarSCAT results for indels and classified them as breakpoint-ambiguous indels, unique-position indels, indels in duplicate and non-duplicate, which were further were used to calculate their proportions. We defined an indel as ‘an ambiguous breakpoint indel’ if its 5’-aligned and 3’-aligned positions differed, and We defined ‘an indel located in a duplicate’ as one where a deletion occurred in a duplicated (or higher order) sequence, or an insertion either generated a novel duplication or extended an existing one. The proportions of in each category were analyzed separately for deletions and insertions.

To illustrate the particularity of ambiguous breakpoint indels, we created a semi-random small indel set for which the total number and size distribution of indels were simulated using the Platinum Genome NA12878 indel set but inserted into the human reference genome GRCh38 at random positions. The sequences of simulated insertions were randomly generated with sequence alphabet ‘A’, ‘T’, ‘G’, and ‘C’. The detection of ambiguous breakpoint indels in the random indel set was performed using a custom script, for which the algorithm for annotating ambiguous breakpoint indels was the same as that of VarSCAT but did not require a VCF file as the input.

**Section S5: Data processing for the analysis of small variants in the STR regions**

In this part of the analysis, we determined STR region variants with one or several STR annotations from VarSCAT. For the 1000 Genomes Project samples, after the VarSCAT annotation was completed, we marked all the variants for each subregion according to the VarSCAT results as either STR SNV, STR deletion, STR insertion, non-STR SNV, non-STR deletion, or non-STR insertion, and we gave each variant an ID. The marked variants were integrated with allele frequencies and individual sample genotype information. We selected the variants that belonged to an individual by choosing variants with the genotypes of the individual that were not reference homozygous (0/0). The marked categories of different types of variants were used to calculate their proportions, and the IDs of variants were used to create a Venn diagram (R [version 3.6.3] [2] package “ggVennDiagram” version 1.2.0) to show variants shared by different superpopulations (Fig 7 in main text).

For Platinum Genome NA12877 and NA12878, and GIAB HG002–HG007, we extracted the numbers of STR SNVs, STR deletions, STR insertions, non-STR SNVs, non-STR deletions, and non-STR insertions from the VarCAT results and then calculated their proportions. For the correlation analysis of the sizes of small indels with STR motif sizes (S4–S9 Figs), we chose small indels spanned only one STR region as follows: 1) indels based on our criteria, as described above; 2) only one STR annotation per indel; and 3) exclusion of indels with multiple STR annotations. Due to the small number of large-sized indels, the analysis was limited to an indel size of 30 bp.

To illustrate how STR regions can affect indel calling, we applied VarSCAT with indel calling results of GATK HaplotypeCaller (GATK HC, v4.0.1.2) and VarScan (v2.4.3) from GIAB HG002 whole exome sequencing (WES) data. The GIAB WES data was produced by Oslo University Hospital, on the Illumina HiSeq 2500 instrument. The sequencing data, which is 150bp paired-end reads and 135× coverage, are archived in Sequence Read Archive with accession SRX1453593 [4]. In total, 6,525 indels with sizes < 50bp are included. The indel calling results of GATK HC and VarScan based on GRCh37 from GIAB NA24385 WES data were previously generated for our another study which about indel calling tool evaluation [5]. The evaluation of indel calling results were performed using hap.py [6] (v0.3.8-17-gf15de4a) with the indel truth set of GIAB HG002 v4.2.1 based on GRCh37. The benchmarking VCF file of GIAB HG002 v4.2.1 GRCh37 and the corresponding high-confidence regions BED file were downloaded from https://ftp-trace.ncbi.nlm.nih.gov/ReferenceSamples/giab/release/AshkenazimTrio/HG002_NA24385_son/NISTv4.2.1/GRCh37/. The indel truth set was produced VCFtools (v0.1.17, --keep-only-indels, --recode) and kept only indels in the benchmarking VCF file. The labels of indel calls (true positives, false positives, and false negatives) were generated by hap.py. We annotated all the indel calls of GATK HC and VarScan using VarSCAT with perfect STR regions of which the criteria and parameter settings were stated in Section S1.

**Codes for Analysis Processes**

The source code of VarSCAT is available at https://github.com/elolab/VarSCAT. The codes we used in our analysis are available at https://github.com/elolab/VarSCAT-analysis and Zenodo https://doi.org/10.5281/zenodo.8079476 [7]. All the plots in the manuscript were created with the R package “ggplot2” (version 3.3.3) [8].

**Reference**

1. Tan A, Abecasis GR, Kang HM. Unified representation of genetic variants. Bioinformatics. 2015;

2. R Development Core Team. R Core Team (2020). R: A language and environment for statistical computing. R Foundation for Statistical Computing, Vienna, Austria. URL https://www.R-project.org/. [Internet]. R Found. Stat. Comput. 2019. p. https://www.R--project.org. Available from: http://www.r-project.org

3. Georgiou Y, Cadeau T, Glesser D, Auble D, Jette M, Hautreux M. Energy accounting and control with SLURM resource and job management system. Lect Notes Comput Sci (including Subser Lect Notes Artif Intell Lect Notes Bioinformatics). 2014. p. 96–118.

4. Zook JM, Catoe D, McDaniel J, Vang L, Spies N, Sidow A, et al. Extensive sequencing of seven human genomes to characterize benchmark reference materials. Sci Data. 2016;3.

5. Wang N, Lysenkov V, Orte K, Kairisto V, Aakko J, Khan S, et al. Tool evaluation for the detection of variably sized indels from next generation whole genome and targeted sequencing data. PLoS Comput Biol. 2022;18.

6. Krusche P, Trigg L, Boutros PC, Mason CE, De La Vega FM, Moore BL, et al. Best practices for benchmarking germline small-variant calls in human genomes. Nat Biotechnol. 2019;37:555–60.

7. Ning Wang, Sofia Khan LE. VarSCAT: A computational tool for sequence context annotations of genomic variants [Internet]. Zenodo; 2022. Available from: https://doi.org/10.5281/zenodo.8079476

8. Wickham H. ggplot2 -Positioning Elegant Graphics for Data Analysis [Internet]. Springer. 2016. Available from: http://link.springer.com/10.1007/978-3-319-24277-4%0Ahttp://ggplot2.org
